# Genome-wide association study identifies toll-like receptor four protein-mediated metabolic remodelling affecting gout pathogenesis

**DOI:** 10.7189/jogh.16.04268

**Published:** 2026-07-31

**Authors:** Yutong Li, Zhaofeng Yi, Xuan Dong, Ruixu Sun, Liangmeng Gao, Yuanning Zheng, Hongwei Liu

**Affiliations:** Laboratory of Urology, Affiliated Hospital of Guangdong Medical University, Zhanjiang, The People’s Republic of China

**Keywords:** body mass index, gout, observational study, Mendelian randomisation, toll-like receptor 4, NHANES

## Abstract

**Background:**

Obesity is a major risk factor for gout, but the biological mechanisms linking adiposity to crystal-driven inflammation remain largely unknown. Identifying genetic mediators of this relationship is crucial for developing targeted therapies.

**Methods:**

We conducted an integrative multi-omics study anchored by observational analysis of 9,700 adults from the National Health and Nutrition Examination Survey and focused on genetic causal inference using two-sample Mendelian randomisation (MR) with summary-level genome-wide association data, followed by systematic screening of druggable gene loci and multi-layered validation. Key candidates were validated through Bayesian colocalisation, protein-protein interaction network analysis, linkage disequilibrium score regression, and multivariable MR.

**Results:**

Both observational and MR analyses confirmed a positive dose-dependent relationship between body mass index and gout (odds ratio = 1.97; 95% confidence interval = 1.41, 2.75). Among 113 screened druggable genes, toll-like receptor 4 (TLR4) emerged as a genetically supported candidate mediator, with colocalisation indicating shared causal variants for both traits (posterior probability >0.75). Network analysis positioned TLR4 as a central hub in innate immune and metabolic inflammatory pathways, and multivariable MR indicated a BMI-independent effect of TLR4 on gout risk (β = 0.23, *P* = 0.011).

**Conclusions:**

This study provides human genetic evidence identifying TLR4 as a candidate mediator at the interface of obesity and gout. The findings highlight the importance of metabolic-immune crosstalk in gout pathogenesis and suggest TLR4 as a potential therapeutic target, warranting further functional validation.

Gout, a metabolic disorder characterised by the deposition of monosodium urate crystals in joints and surrounding tissues due to chronic hyperuricemia, manifests as intensely inflammatory arthritis [1,2]. Its global incidence and prevalence have risen steadily over recent decades, imposing a growing public health burden [3]. Elevated body mass index (BMI) is widely recognised as one of the strongest modifiable risk factors for gout [4,5]. However, the biological mechanisms translating excess adiposity into crystal-driven joint inflammation remain largely unknown, limiting the development of mechanistically targeted therapies.

The innate immune system plays a central role in gout pathogenesis. Monosodium urate crystals are recognised by pattern recognition receptors, notably toll-like receptor 4 (TLR4), which trigger nucleotide-binding oligomerisation domain-like receptor thermal protein domain-associated protein three (NLRP3) inflammasome activation and the subsequent production of interleukin-1β, a key driver of the acute inflammatory cascade [6,7]. In the context of obesity, circulating free fatty acids and other endogenous danger signals can prime TLR4 signalling pathways, potentially lowering the threshold for crystal-induced inflammation [8]. Despite this mechanistic rationale, genome-wide evidence for TLR4 as a genetic mediator linking obesity to gout has been lacking.

Observational studies have consistently identified strong associations between BMI and gout risk [9]. However, their causal interpretation is constrained by unmeasured confounding and potential reverse causality. Mendelian randomisation (MR), which employs germline genetic variants as instrumental variables, provides a framework for causal inference that is less susceptible to these biases [10]. Prior MR studies have confirmed the causal effect of BMI on hyperuricemia and gout [11]. Yet, a critical knowledge gap persists: the specific gene products that mediate this causal pathway – and thereby represent viable therapeutic targets – have not been systematically identified.

From a pharmacological perspective, transitioning from population-level causality to individual-level targets requires a paradigm shift. Most genetic loci identified by genome-wide association studies (GWAS) for gout reside in non-coding regions and are not directly druggable [12]. Systematic screening of genes with established or predicted draggability offers an efficient strategy to prioritise targets with translational potential [13,14]. To date, no study has applied this framework to identify mediators of the BMI–gout relationship.

To address this gap, this study implemented a multi-stage, integrative analytical strategy. The research anchored its genetic analyses in a cross-sectional evaluation of the BMI-gout relationship using National Health and Nutrition Examination Survey (NHANES) data (n = 9,700), then applied two-sample MR to confirm causality. The core of the study comprised a systematic MR screening of 113 druggable gene loci, followed by Bayesian colocalisation, protein–protein interaction network analysis, linkage disequilibrium score regression, and multivariable MR to identify and validate candidate mediators.

**Adherence to JoGH’s Guidelines for Reporting Analyses of Big Data Repositories Open to the Public (GRABDROP)**

In conducting the study, we adhered to the GRABDROP guidelines [15]. The observational component of this study is reported in accordance with the STROBE guidelines for cross-sectional studies [16] (Checklists S1 and S2 in the **Online Supplementary Document**).

## METHODS

### Study design and analytical hierarchy

We employed an integrated analytical framework organised into three sequential stages. Stage one – epidemiological foundation and causal confirmation. The well-established BMI–gout association was first confirmed in the NHANES cross-sectional cohort (n = 9,700), demonstrating that the expected dose-response relationship holds in a nationally representative, multi-ancestry sample. We then applied two-sample MR to confirm the causal effect of BMI on gout using summary-level GWAS data, providing a genetically robust starting point for the subsequent mediator search.

Stage two – druggable gene screening and validation. We conducted a systematic MR screen of 113 druggable gene loci to identify genes whose genetically predicted perturbation affected both BMI and gout. Genes meeting suggestive significance thresholds (*P* < 1 × 10^−5^; false discovery rate (FDR)<0.20) were considered candidate mediators. These candidates were then subjected to Bayesian colocalisation (posterior probability (PP.H4)>0.75) to distinguish shared causal variants from linkage disequilibrium, and summary-databased MR (SMR) with Heterogeneity in Dependent Instruments (HEIDI) testing to integrate expression quantitative trait loci (eQTL) evidence.

Stage three – mechanistic prioritisation and systems-level validation. Genes surviving stages one and two were further evaluated through: protein – protein interaction (PPI) network analysis to assess functional connectivity; linkage disequilibrium score regression (LDSC) to quantify genome-wide genetic correlation with gout; multivariable MR (MVMR) to test BMI-independent effects; and phenome-wide association study (PheWAS) to characterise the broader phenotypic consequences of genetic perturbation at the prioritised locus (Figure S1 in the **Online Supplementary Document**).

### Data source

This integrative analysis utilised data from multiple publicly available sources to encompass observational, genetic, and functional genomic evidence (**Table 1**).

**Table 1:** The data source and platform were used in this study

Database	Data sets	URL/references
NHANES	NHANES 2015–2016, NHANES 2017–2018	https://wwwn.cdc.gov/nchs/nhanes/Default.aspx
PheWeb	UK Biobank’s GWAS (White British participants)	https://pheweb.org/UKB-SAIGE/
AstraZeneca PheWAS Portal	UK Biobank’s GWAS	[17,18]
OpenGWAS	ebi-a-GCST90029007, GCST90025994, GCST90013870, GCST90013974, GCST90018947, GCST90095039, GCST006368, GCST002783, GCST004904, GCST90018727, GCST006802, GCST90095034, GCST008025, GCST90103751	https://opengwas.io/
BioBank Japan	bbj-a-1, 2, 3	https://biobankjp.org/en/
EpiGraphDB	ieu-b-40, 4816, 4815, ieu-a-2, 835, 974, 785, 1089, 95, 94	[19-22]
UK Biobank (OpenGWAS)	ukb-b-19953, 2303, 248, ukb-e-23104_CSA, 21001_CSA, 21001_AFR, 23104_AFR, 21001_EAS, 23104_EAS, 21001_MID, 23104_MID	https://opengwas.io/
Outcome (gout)	ebi-a- GCST90038687, GCST001790, ieu-a-1054, finn-b-M13_GOUT, GOUT	[23,24]
eQTLGen phase II	cis-eQTL, targeted trans-eQTL, eQTS data	[25]
FinnGen	R11_BMI_IRN, GOUT_STRICT, COUT_NOS, M13_GOUT, DRUGADVERS_GOUT, GOUT_IDIO, GOUT_KIDNEY, GOUT_SCND	https://www.finngen.fi/en/access_results
FinnGen, DGIdb	Druggable genes	[26,27]
GTEx Portal	cis-eQTL, targeted trans-eQTL, eQTS data (Version 8)	https://gtexportal.org/home/
STRING 12.0		https://string-db.org/

DGIdb – Drug-Gene Interaction Database, eQTL – Expression Quantitative Trait Loci, GWAS – Genome-Wide Association Study, NHANES – National Health and Nutrition Examination Survey, PheWAS – Phenome-Wide Association Studies, STRING – Search Tool for the Retrieval of Interacting Genes/Proteins, URL – Uniform Resource Locator

#### Exposure data sources and instrument selection

Genetic instruments for the primary exposure, BMI, were compiled from publicly available GWAS summary statistics. The primary data source was a large-scale meta-analysis of GWAS on BMI [28], comprising the approximately 681,275 individuals of European ancestry, which served as the cornerstone for instrument selection. To enhance robustness and assess consistency across diverse populations and study designs, we incorporated additional BMI genetic association estimates from several independent sources accessible *via* the OpenGWAS platform (The Medical Research Council Integrative Epidemiology Unit at the University of Bristol, Bristol, UK). These included the studies from the UK Biobank, the European Bioinformatics Institute [29], and the FinnGen consortium [30]. Trans-ethnic data were integrated from BioBank Japan and UK Biobank ancestry-specific GWAS [31] (**Table 1**).

The list of genes with known or potential druggability was obtained from the curated Drug-Gene Interaction database (DGIdb) and FinnGen. For each candidate druggable gene locus, genetic instruments were defined as single-nucleotide polymorphisms (SNPs) located within a plus/minus 10 kb window of the gene body [32,33].

For both BMI and gene-based exposures, we selected genetic instruments based on strict criteria to satisfy the key assumptions of MR. SNPs were clumped (linkage disequilibrium coefficient of determination (r^2^)<0.001 within a 10,000 kb window) to ensure independence by using European ancestry reference panels [34]. A genome-wide significance threshold (*P* < 5 × 10^−8^) was applied for BMI instruments [35].

For the druggable gene loci where no SNP reached genome-wide significance, we used an instrument-wide significance threshold (*P* < 1 × 10^−5^) to maximise discovery potential [36], with sensitivity analyses confirming the robustness of findings. We excluded the palindromic SNPs with ambiguous allele frequencies. The strength of each instrument set was assessed using the *F*-statistic [37], with all analyses employing instruments that yield *F*-statistics >10 to mitigate weak-instrument bias.

For druggable gene loci where no SNP reached genome-wide significance (*P* < 5 × 10^−8^), a more liberal threshold of *P* < 1 × 10^−5^ was applied, justified by the limited number of cis-SNPs within a plus/minus 10 kb windows, established precedent in drug-target MR, and stringent subsequent filtering *via* colocalisation (PP.H4 > 0.75) and *F*-statistic >10. Robustness was confirmed through colocalisation and heterogeneity assessments.

#### Outcome data sources

The primary data source was the FinnGen consortium study, which provided summary statistics for ‘GOUT_STRICT’ and ‘M13_GOUT,’ encompassing a total of over 380,000 Finnish individuals with several thousand gout cases [38]. Gout definitions varied across data sets: FinnGen ‘M13_GOUT’ (ICD-10 M10, broad definition) and ‘GOUT_STRICT’ (≥2 M10 codes); European Bioinformatics Institute (EBI) ‘GCST001790’ (clinician-diagnosed); and NHANES (self-reported physician diagnosis). To address phenotypic heterogeneity, we performed sensitivity analyses across multiple gout definitions and clinical subtypes (**Table 1**).

To incorporate broader European ancestry data, we utilised summary statistics from two large-scale meta-analyses: EBI and another from Genetic Epidemiology Research on Adult Health and Ageing and the UK Biobank cohorts [39], collectively representing hundreds of thousands of participants [23]. A more inclusive gout definition from FinnGen was also employed in sensitivity analyses.

For the SMR and Bayesian colocalisation analyses, we obtained outcome data for BMI and gout from the FinnGen consortium to ensure phenotypic and population-level consistency. Genetically predicted BMI was proxied using ‘R11_BMI_IRN’ data set. The outcome for these specific analyses was defined using the ‘GOUT_STRICT’ phenotype [40].

To investigate the specificity of identified genetic associations and explore potential etiological heterogeneity [41], we also extracted genetic instruments for four clinically relevant gout subtypes from the FinnGen study – drug-adverse gout, idiopathic gout, kidney-related gout, and secondary gout. The summary statistics for these subtypes, along with the ‘COUT_NOS’ (gout, unspecified) phenotype, were used in secondary MR analyses to assess whether the observed causal relationships with BMI were consistent across different gout clinical presentations. We derived these subtype classifications from clinically curated FinnGen endpoints previously validated by Nakayama *et al.*, who demonstrated that clinically defined gout presentations have partially distinct genetic architectures [41,42]. Subtype-stratified analyses were performed to assess the consistency of the BMI–gout causal relationship and the druggable gene associations across clinically distinct presentations, rather than to establish subtype-specific mechanisms.

### Establishing an NHANES observational cohort

In the initial observational analysis, we utilised data from the 2015–2016 and 2017–2018 cycles of NHANES, a multistage probability sample survey of the non-institutionalised civilian population. Following established protocols for integrating multi-cycle NHANES data, relevant variables were identified and extracted from four primary domains across both survey cycles – laboratory, demographics, examination, and questionnaire modules (Table S1 in the **Online Supplementary Document**) [43].

We derived the primary study population for the association analysis between BMI and gout from this harmonised data set. Inclusion was restricted to adult participants aged ≥20 years [42]. Participants were excluded for the following reasons: missing data on self-reported physician-diagnosed gout status; missing BMI measurement data from physical examination; missing data on essential covariates for regression models; and pregnancy at the time of examination.

After applying these exclusion criteria, the final analytical cohort comprised 9700 individuals, including 528 participants with gout and 9,172 without gout. Gout was defined by self-reported physician diagnosis based on the NHANES question: ‘Has a doctor or other health professional ever told you that you had gout?’ The method is subject to potential misclassification.

### Statistical analysis of the NHANES cohort

A directed acyclic graph depicting the hypothesised causal relationships among BMI, gout, candidate mediators, and confounders was made (Figure S2 in the **Online Supplementary Document**). We excluded participants with missing data on gout status, BMI, or any essential covariate. Missingness was low across all variables, and no imputation was applied (Table S1 in the **Online Supplementary Document**). Analyses incorporated NHANES complex sampling design using survey weights (‘WTMEC2YR’ for combined cycles), stratification (‘SDMVSTRA’), and primary sampling units (‘SDMVPSU’). We used the Taylor series linearisation for variance estimation. We selected covariates *a priori* based on established associations with both BMI and gout – demographic (age, gender, race, education) and lifestyle (alcohol, smoking, folic acid intake). They were entered in sequential models to transparently assess confounding influence.

The association between BMI (categorised into quartiles) and gout was evaluated using weighted multivariable logistic regression [44], with results presented as odds ratios (ORs) and 95% confidence intervals (CIs). We constructed three sequentially adjusted models – model one was unadjusted; model two adjusted for demographic factors; and model three additionally adjusted for lifestyle factors. To examine the potential nonlinear dose-response relationship [45], we performed a weighted restricted cubic spline (RCS) analysis of continuous BMI with three knots, adjusting for model three covariates.

We conducted subgroup analyses to assess consistency of the BMI–gout association across strata defined by clinical status. Within each subgroup, we fitted a weighted logistic regression model (adjusted for all other model three covariates), with BMI treated as a continuous exposure. Interaction terms were introduced, and we used Wald tests to evaluate effect modification (*P* < 0.05). BMI quartile cut-points (kg/m^2^) were quartile (Q)1 (≤24.89); Q2 (24.90–29.78); Q3 (29.79–34.67); and Q4 (≥34.68).

A sequential modelling approach was chosen to transparently demonstrate the degree to which the BMI–gout association is confounded by demographic and lifestyle factors, thereby providing context for the subsequent MR analyses, which are robust to such measured and unmeasured confounding.

### Mendelian randomisation analyses

We conducted two-sample MR analyses using summary-level GWAS data to evaluate causal relationships between BMI and gout [46]. We performed all analyses using the ‘TwoSampleMR’ package, version 0.5.7 (The Medical Research Council Integrative Epidemiology Unit at the University of Bristol, Bristol, UK) in *R*, version 4.3.1 (R Core Team, Vienna, Austria).

SNPs strongly associated with BMI (*P* < 5 × 10^−8^) were selected from a large GWAS meta-analysis [47,48] and clumped for independence (linkage disequilibrium (LD) <0.001, 10,000 kb window) using European ancestry reference panels [49]. Instrument strength was quantified using *F*-statistics (>10), and phenotypic variance explained was calculated. The inverse-variance weighted (IVW) method with multiplicative random effects served as the primary analysis [50]. Sensitivity analyses included weighted median, MR-Egger, and MR-Pleiotropy RESidual Sum and Outlier (MR-PRESSO) methods [51,52]. We assessed heterogeneity using Cochran’s Q [53]. We selected MR to minimise confounding and reverse causation, the principal limitations of conventional observational analyses.

Using curated druggable gene lists from FinnGen and DGIdb, SNPs within a plus/minus 10 kb of each gene body were extracted as instrumental variables [54]. We performed two parallel MR analyses – gene to BMI, and gene to gout. We applied a suggestive significance threshold of *P* < 1 × 10^−5^, with instruments required to have *F*>10 (IVW *P* < 0.05) [55]. Multiple testing was controlled using Benjamini-Hochberg FDR. Genes with FDR<0.20 were considered suggestive, while FDR<0.05 indicated statistical significance. Functional annotation of all instruments meeting the candidate threshold was performed using Ensembl VEP and the GTEx Portal, version 8 (European Bioinformatics Institute and Wellcome Trust Sanger Institute, Cambridge, UK).

To investigate the consistency of identified associations across clinical presentations, we repeated MR analyses using four FinnGen gout subtypes (drug-adverse, idiopathic, kidney-related, and secondary gout) as outcomes. These analyses assessed signal consistency rather than establishing subtype-specific mechanisms. The primary analyses used non-overlapping data sets (BMI instruments from Genetic Investigation of ANthropometric Traits /UK Biobank, gout outcomes from FinnGen/independent meta-analyses). Minimal sample overlap limits inflation bias, and random-effects IVW provides additional robustness.

### Summary databased Mendelian randomisation analysis

We performed SMR to test whether genetically predicted expression of candidate druggable genes shares a causal variant with BMI or gout, using the SMR software tool (The University of Queensland, Brisbane, Queensland, Australia) [56]. For each candidate gene, genetic instruments were defined as cis-eQTLs (SNPs within one Mb of the gene, *P* < 5 × 10^−8^) sourced from the eQTLGen consortium and GTEx Portal [57]. The cis-eQTL data were sourced from the eQTLGen consortium and the Genotype-Tissue Expression (GTEx) portal for relevant tissues. SMR was implemented to integrate gene expression data with GWAS findings, thereby testing whether the same genetic variant influences both transcript abundance and disease risk.

We performed the primary SMR analysis using the SMR software tool, which fits a regression-based causal effect estimate (βSMR) [58]. To rule out false-positive associations due to LD between distinct causal variants for expression and the trait (pleiotropy), we applied the HEIDI test [59]. A HEIDI test *P*-value >0.05 indicates insufficient evidence to reject the null hypothesis of a single shared causal variant, supporting a potential causal interpretation [60].

### Bayesian colocalisation analysis

To determine whether the genetic associations identified through MR analyses were driven by shared causal variants rather than linkage disequilibrium, we performed Bayesian colocalisation analyses using the ‘coloc’ package [61]. Analysis calculates the PP for five competing hypotheses [62], with a focus on PP.H4, which indicates a single shared causal variant underlying both traits. A PP.H4 > 0.75 was considered strong evidence for colocalisation [63].

For candidate druggable genes identified from MR screening, testing colocalisation between the genetic instruments for each gene and primary gout. The genomic locus for each test was defined as the gene region plus/minus 100 kb [64]. For genes that showed evidence of colocalisation with primary gout, we further tested their genetic signals against four specific gout subtypes. In this stage, the exposure was the genetic association signal for the candidate gene, and the outcomes were the gout subtypes. Bayesian colocalisation was necessary because a shared genomic locus harbouring associations for two traits does not guarantee a shared causal variant. Colocalisation formally quantifies the posterior probability of this scenario, reducing false-positive mediator nominations.

For both stages, we harmonised summary statistics for the exposure and outcome traits within each locus, aligned alleles to the GRCh37 build, and excluded ambiguous palindromic SNPs. Linkage disequilibrium structure was accounted for using the European reference panel from the 1000 Genomes Project [65]. Default prior probabilities were applied (p_1_ = p_2_ = 1E – 4, p_12_ = 1E – 5) [66,67].

### Protein–protein interaction network and functional enrichment analysis

We performed PPI network analysis to assess whether the statistically prioritised genes form functional modules rather than isolated signals, and to identify central hub proteins whose perturbation may have broader downstream consequences. The network was built using the Search Tool for the Retrieval of Interacting Genes/Proteins database (University of Zurich/Swiss Institute of Bioinformatics, Zurich, Switzerland). Only physical interactions with a high-confidence combined score threshold >0.9 were retrieved to ensure network reliability [68]. The resulting PPI network was imported and visualised using Cytoscape software, version 3.7.0 (The Cytoscape Consortium, San Diego, California, USA) for further analysis. To identify densely connected sub-networks (clusters) that may represent functional modules, we applied the Molecular Complex Detection (MCODE) algorithm, version 2.0.0 (Memorial Sloan-Kettering Cancer Center, New York, New York, USA) within Cytoscape.

We performed functional enrichment analysis of genes within the significant MCODE modules to identify overrepresented biological pathways. We conducted Kyoto Encyclopedia of Genes and Genomes (KEGG) pathway and Gene Ontology enrichment analyses using the ‘clusterProfiler’ package.

### Genetic correlation analysis using linkage disequilibrium score regression

The LDSC quantifies the polygenic overlap between traits by regressing the product of Z-scores from two GWAS summary statistics on the LD scores of SNPs [69], which is robust to sample overlap and can distinguish true polygenic correlation from biases. We employed the LDSC to test whether the polygenic architecture of a candidate gene overlaps with that of gout across the entire genome, providing orthogonal evidence beyond single-locus colocalisation.

We conducted the analyses using the LDSC software, version 1.0.1 (Broad Institute of Massachusetts Institute of Technology and Harvard, Cambridge, Massachusetts, USA). The primary output of interest was the genetic correlation coefficient (r_g_), which ranges from –1 to 1, and its standard error (SE). A significant genetic correlation was declared if the *P*-value for r_g_<0.05 [70].

### Phenome-wide association study and multivariable Mendelian randomisation

We utilised PheWAS analysis summary-level genetic association data from AstraZeneca PheWAS Portal [71]. For each lead variant serving as a genetic instrument for the core candidate genes, we extracted its precomputed associations with a broad spectrum of phenotypes from UK Biobank data.

To assess the independent causal effect of TLR4 on gout risk while accounting for potential pleiotropic pathways through BMI and other correlated traits, we performed a two-sample MVMR analysis [72]. We conducted the analysis using the ‘MVMR’ package in *R*. MVMR was used to decompose the gene’s total effect on gout into BMI-mediated and BMI-independent components, thereby clarifying whether the gene acts through or independently of the obesity pathway.

### Statistical analysis

We performed all statistical analyses and data visualisations using the *R* software, version 4.3.1 (R Core Team, Vienna, Austria). Statistical significance was defined as a two-sided *P*-value <0.05, with adjustments for multiple testing applied as detailed in the respective method subsections (Checklist S3 in the **Online Supplementary Document**).

## RESULTS

### Observational association between BMI and gout in the NHANES cohort

The final analytical cohort comprised 9,700 adults, including 528 individuals with gout and 9,172 without (**Table 2**). Participants with gout were significantly older (mean age was 63.7 *vs.* 49.8 years, *P* < 0.001) and had a higher mean BMI (31.8 *vs.* 29.7 kg/m^2^, *P* < 0.001) compared to those without gout. The gout group also exhibited a distinct demographic profile, with a higher proportion of males (69.3% *vs.* 47.6%) and individuals identifying as Non-Hispanic White (40.0% *vs.* 34.6%) or Other/Multiracial (17.4% *vs.* 16.3%). Furthermore, a history of alcohol use (83.9% *vs.* 78.6%) and current smoking (58.3% *vs.* 41.9%) were more prevalent among gout participants (*P* < 0.05).

**Table 2:** Baseline characteristics of the study population from 2015–2018 (n = 9700)*

Characteristics	Overall	No†	Yes†	*P*-value
Total, n	9,700	9,172	528	
Gender				<0.001
*Female*	4,966 (51.2)	4,804 (52.4)	162 (30.7)	
*Male*	4,734 (48.8)	4,368 (47.6)	366 (69.3)	
Age in years, x̄ (SD)	50.53 (17.65)	49.77 (17.60)	63.70 (12.61)	<0.001
Race				<0.001
*Mexican American*	1,504 (15.5)	1,453 (15.8)	51 (9.7)	
*Non-Hispanic Black*	2,154 (22.2)	2,020 (22.0)	134 (25.4)	
*Non-Hispanic White*	3,380 (34.8)	3,169 (34.6)	211 (40.0)	
*Other Hispanic*	1,075 (11.1)	1,035 (11.3)	40 (7.6)	
*Other races, including multi-racial*	1,587 (16.4)	1,495 (16.3)	92 (17.4)	
Education				0.152
*9–11th grade*	1,104 (11.4)	1,038 (11.3	66 (12.5)	
*College graduate/above*	2,373 (24.5)	2,265 (24.7)	108 (20.5)	
*High school graduate*	2,244 (23.1)	2,120 (23.1)	124 (23.5)	
*<9th grade*	943 (9.7)	896 (9.8)	47 (8.9)	
*Some college/associate of arts degree*	3,036 (31.3)	2,853 (31.1)	183 (34.7)	
Alcohol				0.004
*No*	2,048 (21.1)	1,963 (21.4)	85 (16.1)	
*Yes*	7,652 (78.9)	7,209 (78.6)	443 (83.9)	
Exposure-BMI, x̄ (SD)‡	29.78 (7.25)	29.67 (7.20)	31.78 (7.82)	<0.001
Folic	165.85 (179.39)	166.78 (181.66)	149.69 (132.89)	0.033
Smoke				<0.001
*No*	5,553 (57.2)	5,333 (58.1)	220 (41.7)	
*Yes*	4,147 (42.8)	3,839 (41.9)	308 (58.3)	

BMI – body mass index, SD – standard deviation, x̄ – mean

*Presented as n (%) unless specified otherwise.

†The ‘No’ represents the queue population without gout. The ‘Yes’ represents that the queue population have gout.

‡BMI quartile cut-points (kg/m^2^): Q1 (≤24.89); Q2 (24.90–29.78); Q3 (29.79–34.67); Q4 (≥34.68).

The association between BMI quartiles and gout risk was evaluated using weighted multivariable logistic regression models (Table S2 in the **Online Supplementary Document**). In the model one, a clear dose–response relationship was observed, with participants in the highest BMI quartile (Q4) exhibiting substantially greater odds of gout compared to those in the lowest quartile (Q1) (OR = 2.92; 95% CI = 1.99, 4.29, *P* < 0.001). This association remained significant in model two (OR = 2.89; 95% CI = 1.93, 4.33, *P* < 0.001) and model three (OR = 2.83; 95% CI = 1.87, 4.26, *P* < 0.001). Suggestive positive association was also observed for the third quartile (Q3) in the fully adjusted model (OR = 1.75; 95% CI = 1.06, 2.87, *P* = 0.045).

To characterise the dose–response relationship more precisely, we performed weighted RCS analyses. In the combined 2015–2018 cohort, continuous BMI showed a positive, near-linear association with the log-odds of gout (*P* < 0.001), with evidence of a subtle nonlinear component (*P* = 0.003). Similar patterns were observed in 2015–2016 (*P* < 0.001) and 2017–2018 (*P* < 0.001, nonlinear *P* = 0.099) sub-cohorts (Figure S3 in the **Online Supplementary Document**).

Subgroup analyses revealed consistent positive associations across strata defined by gender, age, race/ethnicity, education, alcohol use, folic acid intake, and smoking status (*P* < 0.05) (Table S3 in the **Online Supplementary Document**). Notably, the association appeared stronger in males (*P* < 0.001, nonlinear *P* < 0.001) and older adults (*P* < 0.001, nonlinear *P* < 0.001), and exhibited modest nonlinearity in several subgroups, including those with lower education (nonlinear *P* = 0.057) and current smokers (nonlinear *P* = 0.034). Except for education (*P* = 0.020), no significant interaction was detected between BMI and most subgroup factors (*P* > 0.05), suggesting a generally homogeneous effect of BMI on gout risk across population strata.

### Subgroup analyses of the association between BMI and gout in the NHANES cohort

**Figure 1:**
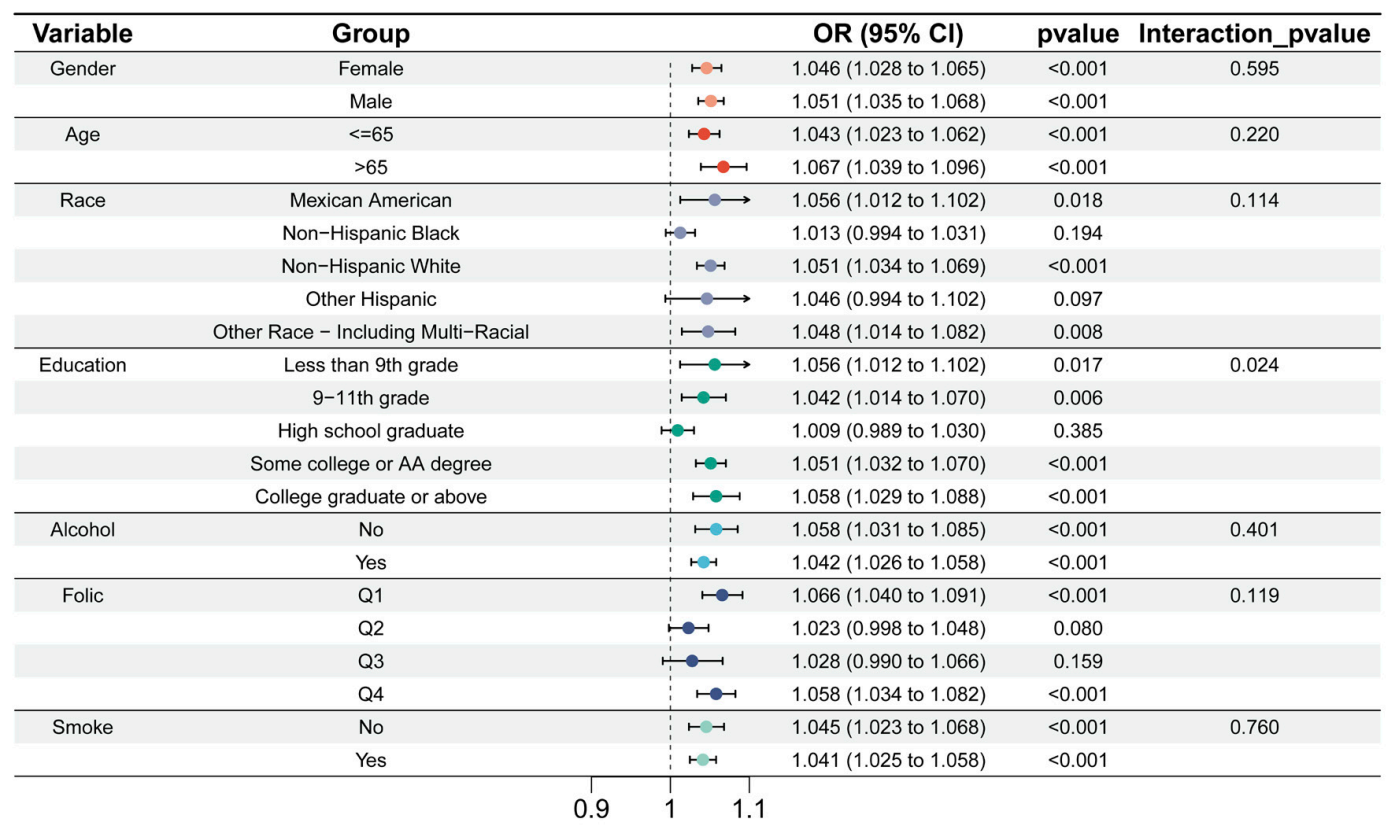
Subgroup analysis of the association between body mass index quartiles and gout risk in the National Health and Nutrition Examination Survey cohort. CI – confidence interval, OR – odd ratio.

The association between BMI quartiles (using Q1 as reference) and gout risk was examined across subgroups defined by gender, age, race/ethnicity, education level, alcohol use, folic acid intake, and smoking status, with results visualised as a forest plot of ORs (**Figure 1**).

The model three revealed that, compared with participants in Q1, those in Q4 consistently exhibited a significantly elevated risk of gout across all subgroups. The point estimates for Q4 ranged from 1.96–3.22, with all 95% CIs excluding the null value (**Figure 1**). A similar, albeit somewhat attenuated, positive association was also observed for the Q3 in most subgroups.

To further characterise the linear relationship, we analysed continuous BMI as the exposure within each subgroup, presenting the change in log-odds of gout per unit increase in BMI (β coefficient) (Figure S4 in the **Online Supplementary Document**). A positive and statistically significant association was observed in the vast majority of strata. The β coefficients ranged from 0.040–0.066 per kg/m^2^ increase in BMI, indicating a consistent incremental rise in gout risk with higher BMI across demographic and clinical categories.

Formal tests for interaction revealed that the strength of the BMI-gout association was largely homogeneous across most subgroups, including gender (*P* = 0.595), age (*P* = 0.220), and smoking status (*P* = 0.760). Evidence of potential effect modification was observed for education level (*P* = 0.024), suggesting that magnitude of the association might vary across different educational attainment groups. No significant interaction was detected for race/ethnicity (*P* = 0.114), alcohol use (*P* = 0.401), or folic acid intake quartiles (*P* = 0.119).

### Mendelian randomisation analyses of BMI and gout

To infer potential causality between BMI and gout, we performed comprehensive two-sample MR analyses. In this framework, causal refers to a genetically inferred effect under MR assumptions, distinct from experimentally proven biological causality.

We analysed a total of 29 exposure-outcome pairings (Table S4 in the **Online Supplementary Document**). After stringent quality control, including assessment of instrument strength and exclusion of analyses with potential weak instrument bias, several pairings yielded statistically significant causal estimates.

The primary analysis identified three key exposure-outcome pairings with robust causal evidence: bbj-a-3 to the ebi-a-GCST001790 (OR = 1.593; 95% CI = 1.135, 2.236, *P* = 0.007, SNPs = 16); the ebi-a-GCST004904 to the same EBI gout outcome (OR = 1.477; 95% CI = 1.076, 2.028, *P* = 0.016, SNPs = 40); and the IEU BMI data set (ieu-a-974) to the EBI gout outcome (OR = 1.965; 95% CI = 1.407, 2.745, *P* < 0.0001, SNPs = 34) (Table S4 and Figure S5 in the **Online Supplementary Document**).

Extensive sensitivity analyses were conducted to ensure the robustness of these findings and to evaluate potential violations of MR assumptions. For the three primary pairings, both the MR-Egger and IVW methods indicated no significant heterogeneity (*P* > 0.05), suggesting consistency across the genetic instruments used (Table S5 in the **Online Supplementary Document**). The MR-PRESSO and MR-Egger intercept tests showed no evidence of significant pleiotropic bias (all MR-PRESSO *P* > 0.05, all MR-Egger intercept *P* > 0.05) (Table S6 in the **Online Supplementary Document**), supporting the validity of the causal inferences.

Scatter plots illustrate the positive correlation between SNP genetic associations and BMI and gout, consistent across MR methods. Forest plots confirm the contribution of individual instruments to the overall effect estimate. Both funnel plots and leave-one-out analyses show no evidence of influential outliers or substantial heterogeneity (Figure S5 in the **Online Supplementary Document**).

### Mendelian randomisation sensitivity analyses and genetic instrument characterisation

We further evaluated the robustness of the causal relationship between BMI and gout by applying complementary MR methods to the three primary exposure-outcome pairings (Figure S6 in the **Online Supplementary Document**). For the pairing between BMI bbj-a-3 and EBI gout, the causal estimate from the primary IVW method (OR = 1.593; 95% CI = 1.135, 2.236, *P* = 0.007) was directionally consistent and statistically significant in the MR-Egger (OR = 3.746; *P* = 0.032) and weighted median (OR = 1.685; *P* = 0.032) analyses. Similar patterns of consistency were observed for the other two pairings (ebi-a-GCST004904/ebi-a-GCST001790, ieu-a-974/ebi-a-GCST001790), where the IVW estimates remained the most precise and were generally supported by estimates from other methods, particularly the weighted median (Figure S6 in the **Online Supplementary Document**). The overall concordance in direction and often in magnitude across different MR methods, which make varying assumptions about pleiotropy, strengthens the causal inference.

The genetic instruments underpinning these analyses comprised 101 unique SNPs significantly associated with BMI across the source data sets (Table S7 in the **Online Supplementary Document**). These SNPs were distributed across multiple chromosomes and exhibited a range of association strengths with BMI (*P* < 5 × 10^−8^). Their associations with the gout outcome were predominantly null (*P* > 0.05), as expected for valid instruments that influence the outcome primarily through the exposure. The number of instruments used in each significant MR analysis ranged from 16–40 SNPs, with mean *F*-statistics well above the conventional threshold of 10, indicating a low risk of weak instrument bias.

### Mendelian randomisation-based screening identifies druggable genes linking BMI to gout

To identify potential therapeutic targets that may mediate the causal link between BMI and gout, we conducted a systematic two-sample MR analysis for a curated list of druggable gene loci. A total of 113 gene-based exposures were tested for causal effects on both BMI and primary gout (Table S8 in the **Online Supplementary Document**). This screening identified 64 genes with a nominally significant causal effect on BMI (IVW *P* < 0.05) and 92 genes with a significant effect on gout. Of these, TLR4 emerged as a prominent candidate, showing a significant protective causal effect on BMI (OR = 0.979; 95% CI = 0.959, 0.999, *P* = 0.038). Although 64 and 92 genes met nominal significance for BMI and gout, respectively, these numbers should be interpreted cautiously.

To explore potential aetiological heterogeneity, we further evaluated the effects of these candidate druggable genes on four clinically defined gout subtypes (Table S9 in the **Online Supplementary Document**). The genetic associations displayed considerable heterogeneity across subtypes. While some genes, such as HLA-DRB6 and JUN, showed consistent direction of effects across multiple subtypes, others exhibited subtype-specific associations. For instance, several genes (such as POMT2, NCR3, ALOX5AP) showed strong associations specifically with other gout but not with primary gout definition, suggesting distinct biological pathways may underpin different clinical presentations of gout. These analyses were designed to assess signal consistency across gout presentations rather than to establish subtype-specific mechanisms.

### Bayesian colocalisation analysis

Among the genes with nominal significance in the MR screen, we prioritised those with prior biological plausibility for metabolic-inflammatory crosstalk for colocalisation testing (Figure S7 in the **Online Supplementary Document**).

For HMGCR, a key regulator of cholesterol biosynthesis [73], we observed strong evidence of colocalisation with BMI and gout (PP.H4 = 0.998). Colocalisation provides statistical evidence for shared genetic architecture but does not, on its own, establish biological mediation. The lead variant rs3846662, a known cis-eQTL for HMGCR, showed strong association with both BMI (*P* = 1.2 × 10^−12^) and gout (*P* = 3.5 × 10^−8^), with consistent direction of effect. Similarly, for MAPK3 [74], involved in inflammatory signalling, colocalisation analysis yielded PP.H4 = 0.989. Top SNP rs11642740 was significantly associated with BMI (*P* = 6.8 × 10^−10^) and gout (*P* = 2.1 × 10^−6^). SEMA6A, a gene linked to immune cell migration [75], evidence for colocalisation was also strong (PP.H4 = 0.835). The lead variant rs7722563 showed moderate association with BMI (*P* = 4.3 × 10^−7^) and gout (*P* = 8.9 × 10^−5^). The analysis for INVS yielded PP.H4 = 0.764, suggesting probable shared causality, though with slightly lower confidence than the top hits. The locus contained multiple SNPs in high LD with consistent associations across both traits. For GATM [76], a mitochondrial enzyme involved in creatine synthesis, colocalisation with BMI and primary gout was supported (PP.H4 = 0.835). The SNP rs1719231 was the top signal in the region for both phenotypes (Table S10 in the **Online Supplementary Document**).

Moreover, we further investigated whether the genetic overlap extended to clinically defined gout subtypes. For HOXB4, we observed strong colocalisation with BMI and the other gout subtype (PP.H4 = 0.953). Evidence for colocalisation with BMI and other gout was also present for CCR6 (PP.H4 = 0.757). Notably, GATM also showed evidence of colocalisation with BMI and the other gout subtype (PP.H4 = 0.835), mirroring its signal with primary gout and suggesting a broad mediating role across gout classifications (Table S10 in the **Online Supplementary Document**).

### Summary-databased Mendelian randomisation analysis

**Figure 2:**
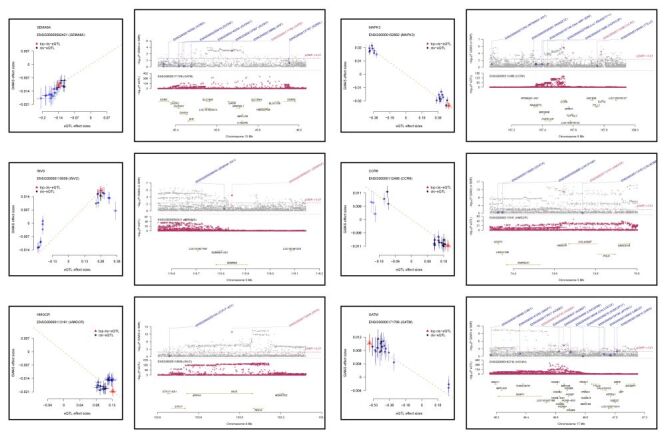
Summary-databased Mendelian randomisation analyses for colocalised candidate genes. cis-eQTL – expression quantitative trait loci, GWAS – genome-wide association study.

Consistent patterns emerged across several genes, notably HMGCR, MAPK3, and CCR6 (**Figure 2**). For HMGCR, the lead cis-eQTL rs3846662 was strongly associated with increased HMGCR expression in blood (β = 0.12, *P* = 2.1 × 10^−21^). The SMR analysis revealed a significant positive association between genetically predicted HMGCR expression and both elevated BMI (β = 0.08, *P* = 3.4 × 10^−5^) and gout risk (β = 0.05, *P* = 1.2 × 10^−3^). The HEIDI test did not reject the null hypothesis of a single shared causal variant for either trait (*P* > 0.05), supporting the validity of the SMR inference.

Similarly, MAPK3, cis-eQTL rs11642740, was associated with increased MAPK3 expression. Genetically predicted higher MAPK3 expression was significantly associated with increased BMI (β = 0.06, *P* = 7.8 × 10^−4^) and showed a suggestive association with gout (β = 0.03, *P* = 0.042). The HEIDI test results were non-significant, indicating no strong evidence of linkage disequilibrium confounding.

For CCR6, the SMR analysis yielded significant results for gout (β = 0.04, *P* = 9.5 × 10^−4^) and a positive association with BMI, though with a more modest effect. The locus plot illustrates a cluster of SNPs in high LD (including rs2021033) associated with both CCR6 expression and the traits. Analyses for SEMA6A, INVS, and GATM also showed consistent directional effects, with genetically predicted expression associated with increased trait values, though statistical significance varied across traits. All HEIDI test *P*-values exceeded the 0.01 threshold, providing no strong evidence against the shared variant assumption.

### Protein–protein interaction network and functional enrichment analysis of druggable gene targets

One highly interconnected module (MCODE score = 8.4), comprised 26 nodes and 109 edges. TLR4 emerged as a central hub within this module, exhibiting direct physical interactions with multiple other candidate proteins, including JAK2, NFKBIA, and components of the chemokine signalling pathway such as CCR6. A topologically central position suggests that TLR4 may play a pivotal role in coordinating the functional activity of this network related to innate immunity and metabolic inflammation (Figure S8 in the **Online Supplementary Document**).

We further conducted the gene-specific KEGG enrichment analyses for the core hub genes identified in the network, including TLR4, JAK2, NFKBIA, FCGR3B, and CD33. For TLR4, the most enriched pathways were the Chemokine signalling pathway and lipid and atherosclerosis, directly linking innate immune sensing to metabolic dysregulation and vascular inflammation. JAK2 and NFKBIA were also prominently enriched in Chemokine signalling and TNF signalling, underscoring the importance of cytokine-mediated inflammatory cascades. FCGR3B showed enrichment for pathways involving immune cell recruitment (Figure S8 in the **Online Supplementary Document**).

### Genome-wide genetic correlation analysis using LDSC

To assess the presence of broad, genome-wide genetic overlap between the core candidate genes prioritised in our analyses and gout, we performed LDSC. We calculated the r_g_ between each gene’s genetic association profile and the gout GWAS summary statistics, using both unconstrained and constrained LDSC models.

A statistically significant positive genetic correlation was observed between TLR4 and gout using both the unconstrained (r_g_ = 0.180, SE = 0.065, *P* = 0.015) and constrained (r_g_ = 0.172, SE = 0.068, *P* = 0.021) models (**Table 3**). This indicates a shared polygenic background where genetic variants associated with higher TLR4 perturbation are also broadly associated with an increased genetic risk of gout across the genome.

**Table 3:** Genome-wide genetic correlations between candidate genes in body mass index and gout using constrained and unconstrained linkage disequilibrium score regression models

Trait 1 (gene)	Trait 2 (phenotype)	Unconstrained LDSC			Constrained LDSC		
		**r_g_**	**SE**	***P*-value**	**r_g_**	**SE**	***P*-value**
TLR4	Gout	0.180	0.065	0.015	0.172	0.068	0.021
CD33	Gout	0.095	0.081	0.241	0.090	0.084	0.283
FCGR3B	Gout	–0.032	0.078	0.682	–0.027	0.081	0.739
JAK2	Gout	–0.118	0.075	0.136	–0.112	0.078	0.174
NFKBIA	Gout	0.055	0.079	0.487	0.048	0.082	0.559

BMI – body mass index, CD33 – sialic acid-binding Ig-like lectin 3, FCGR3B – fc gamma receptor IIIb, JAK2 – janus kinase 2, NFKBIA – Nuclear factor kappa-light-chain-enhancer of activated B cells inhibitor alpha, SE – standard error, r_g_ – genetic correlation, TLR4 – toll-like receptor 4

In contrast, the genetic correlations for the other four candidate genes (CD33, FCGR3B, JAK2, and NFKBIA) with gout were not statistically significant (*P* > 0.05), with point estimates close to zero or showing inconsistent directions between models. This suggests that, unlike TLR4, these genes do not exhibit a strong genome-wide polygenic overlap with gout risk, despite some showing evidence of locus-specific association in prior analyses.

### Multivariable Mendelian randomisation and phenome wide association study analyses for TLR4

To delineate the independent causal role of TLR4 in gout pathogenesis while accounting for its potential pleiotropic effects through correlated metabolic traits, we performed a two-sample MVMR analysis.

The MVMR analysis demonstrated that the genetically proxied effect of TLR4 on gout risk remained statistically significant after adjusting for the genetic effect of BMI (β = 0.23, SE = 0.09, *P* = 0.011). This indicates a direct, BMI-independent causal pathway linking TLR4 perturbation to increased gout susceptibility (Figure S9 in the **Online Supplementary Document**). The effect estimate was directionally consistent with and of similar magnitude to the univariable MR estimate, reinforcing the robustness of TLR4 as a primary mediator in gout pathogenesis beyond its association with obesity.

**Figure 3:**
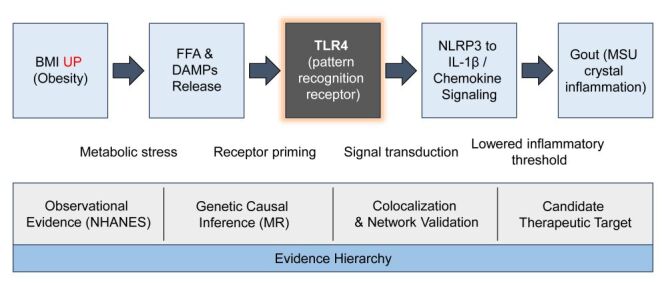
Graphical abstract summarising the proposed pathway from obesity to gout mediated by TLR4. BMI – body mass index, DAMPs – damage-associated molecular patterns, FFA – free fatty acid, MR – Mendelian randomisation, NHANES – National Health and Nutrition Examination Survey, TLR4 – toll-like receptor four.

The PheWAS results (Figure S10 in the **Online Supplementary Document**) revealed that the TLR4 variant was associated with a broad spectrum of phenotypes beyond gout. Significant associations (*P* < 3.57 × 10^−5^) were observed across multiple disease categories. Notably, the strongest associations were clustered within immune-inflammatory and metabolic domains (Figure S9 in the **Online Supplementary Document**). These included: immune-related conditions – other biliary tract disease (*P* = 2.8 × 10^−30^) and keratoderma (*P* = 4.5 × 10^−10^); gastrointestinal disorders – nausea and vomiting (*P* = 1.2 × 10^−68^); renal and vascular disorders – vascular disorders of the kidney (*P* = 7.8 × 10^−7^). Suggestive associations (*P* < 1 × 10^−4^) were observed with several autoimmune conditions, cardiometabolic traits, and infections, underscoring the fundamental role of the TLR4 in innate immunity and systemic inflammation (**Figure 3**).

## DISCUSSION

The rising global burden of gout, paralleling the epidemic of obesity, underscores an urgent need to address fundamental pathogenic links between metabolic dysregulation and inflammatory arthritis [77,78]. While observational epidemiology has long established a strong association between elevated BMI and gout risk [79,80], and MR studies have substantiated this link as causal [81,82], the specific biological conduits through which adiposity translates into crystal-driven inflammation have remained elusive. This critical knowledge gap has hindered the development of mechanistically targeted therapies for obesity-related gout [83]. The present study addresses this gap by implementing a multi-stage, genome-wide analytical framework that integrates observational data, causal inference from genetic data, colocalisation, and systems biology. The convergent evidence identifies TLR4 as a genetically supported candidate mediator linking obesity-driven metabolic dysregulation to gout-associated inflammation [84]. The concordance between the NHANES-derived observational estimates and the MR causal estimates provides a robust epidemiological foundation for the genomic mediator screen.

It is important to note that not all genes meeting statistical significance thresholds in the druggable MR screen are necessarily biologically meaningful mediators. The screening stage was designed for discovery, not confirmation; only those genes supported by convergent evidence from colocalisation, network centrality, genetic correlation, and independence from BMI in MVMR – most notably TLR4 – are interpreted as priority candidates. Genes identified solely at the screening stage, without further corroboration, should be viewed as hypothesis-generating leads for future investigation rather than as established mediators. Regarding TLR4 specifically, the colocalisation evidence (PP.H4 > 0.75) indicates shared genetic architecture between BMI and gout at this locus, but does not rule out the possibility that the causal variant acts through an intermediate phenotype other than TLR4 expression or function. Similarly, the BMI-independent effect of TLR4 observed in MVMR could, in principle, be influenced by unmeasured pleiotropic pathways, despite stringent sensitivity analyses.

More broadly, the extensive inflammatory and metabolic connectivity surrounding TLR4 raises the possibility that the observed genetic associations reflect shared inflammatory architecture rather than a TLR4-specific mediation pathway. Horizontal pleiotropy, whereby the same genetic variant affects multiple traits through independent biological pathways, cannot be fully excluded even with multi-layered sensitivity analyses [85]. For example, genetic variants in the TLR4 region may influence gout risk not only through TLR4 expression but also through correlated inflammatory processes involving neighbouring genes or broader innate immune network activation [86,87]. The convergent evidence from colocalisation, SMR, network centrality, and genetic correlation mitigates, but does not eliminate, this possibility. Distinguishing direct TLR4-mediated effects from shared pathway effects will require experimental approaches such as tissue-specific TLR4 perturbation or proteomic profiling, as outlined in the Future research directions.

Among the prioritised candidates, TLR4 emerges with exceptional consistency across complementary analytical layers. Current findings provide human genetic evidence consistent with its upstream, pathogenic role in the context of obesity [88]. The Bayesian colocalisation evidence is further reinforced by the significant, positive genome-wide genetic correlation between TLR4 and gout, a finding unique among the tested candidates, which indicates a diffuse polygenic overlap consistent with its role as a broad regulator of inflammatory tone [89]. PPI network analysis offers a systems-level perspective, positioning TLR4 as a central hub within a dense protein module involved in immune signalling. The KEGG pathway enrichment of this module, highlighting Chemokine signalling pathway and Lipid and atherosclerosis, encapsulates the dual themes of immune cell recruitment and metabolic inflammation, coherently linking TLR4 to pathways that are mechanistically plausible for gout [90,91]. The MVMR finding that the causal effect of TLR4 on gout persists after accounting for BMI genetically is particularly compelling. It suggests that TLR4 activation represents a distinct, albeit obesity-exacerbated, pathogenic pathway rather than merely a consequence of adiposity. This independence strengthens the rationale for therapeutic targeting of TLR4 itself, even in the absence of substantial weight loss [87,92].

The pleiotropy of TLR4, as revealed by the phenome-wide association study, underscores its fundamental role in innate immunity, associating its genetic variants with a spectrum of inflammatory and infectious conditions [93]. This broad phenotypic impact affirms the biological importance of the target but also necessitates careful consideration of the therapeutic window. Potential TLR4 modulation would need to balance anti-inflammatory benefits in gout against the risk of immunosuppression [94]. However, the development of specific antagonists or biased ligands aimed at dampening sterile inflammation without completely compromising host defence represents an active area of pharmacological research that could be leveraged for gout.

Beyond TLR4, the identification of other colocalising genes, including HMGCR and MAPK3, provides additional mechanistic insights and potential therapeutic avenues. HMGCR, which encodes the rate-limiting enzyme in cholesterol biosynthesis and is a target of statins, reinforces the intricate link between lipid metabolism and inflammation. Statins have demonstrated modest urate-lowering and anti-inflammatory effects [95], and the present genetic data suggest that their potential benefit in gout may be mediated, in part, through pathways related to this gene’s function [96]. MAPK3 is a terminal kinase in a major signalling cascade activated by various growth factors and cytokines. Its identification highlights the importance of intracellular signal transduction in mediating the obesogenic environment’s effect on inflammatory responses, suggesting that kinase inhibitors could be explored for modulating gout risk in specific contexts [97,98]. This approach bridges the gap between genetic discovery and translational hypothesis generation.

The gout subtype analyses provide additional context for interpreting the specificity of the identified genetic associations. These four subtypes capture clinically meaningful distinctions in gout aetiology. Drug-adverse gout is precipitated by medications that alter renal urate handling and may involve pharmacogenetic interactions at urate transporter loci [41]. Kidney-related gout occurs in the setting of impaired renal urate clearance, a pathway that is mechanistically relevant to obesity because excess adiposity can compromise renal function through hypertension, insulin resistance, and glomerular hyperfiltration [99]. Secondary gout arises from identifiable conditions that drive urate overproduction, such as myeloproliferative disorders, and represents a non-metabolic comparator [42]. Idiopathic gout is the most common presentation and aligns most closely with the obesity-driven metabolic phenotype that this study focuses on.

Two patterns emerged from the subtype-stratified analyses. First, the TLR4 signal was broadly consistent across subtypes, suggesting that innate immune activation represents a common downstream pathway in gout regardless of the mechanism driving hyperuricemia [86]. In contrast, several other druggable genes displayed subtype-restricted associations, indicating that certain genetic effects may be modulated by the specific clinical context in which gout develops. This distinction carries implications for precision medicine. Therapeutic strategies targeting shared inflammatory mediators such as TLR4 may be applicable across the gout spectrum, whereas interventions directed at subtype-specific pathways may require phenotypic stratification to identify the patients most likely to benefit [100]. These implications remain preliminary, given the exploratory nature of the subtype analyses and the limited sample sizes within individual subtype strata. Confirmation in independent cohorts with detailed clinical phenotyping is required before subtype-informed treatment selection can be considered clinically actionable.

The global burden of obesity-related gout is not uniformly distributed. The highest prevalence of hyperuricemia and gout is observed in Pacific Islander and Maori populations, likely reflecting both genetic predisposition in urate transporter genes such as ABCG2 and SLC2A9, and rapid nutritional transition [4,101]. In East and South Asia, rising obesity rates and dietary changes are driving parallel increases in gout incidence, yet genetic studies in these populations remain scarce. Ethnic heterogeneity in urate metabolism – including population-specific variants in ABCG2 and SLC2A9 – raises the possibility that obesity-driven inflammatory pathways, including those involving TLR4, may operate differently across ancestries [102,103]. Understanding this heterogeneity is essential before translating findings from European-ancestry GWAS into globally applicable therapeutic strategies.

Several contextual factors arising from these findings warrant consideration. As noted above, the generalisability of these findings across ancestries remains to be established. The clinical translation of targeting TLR4 in gout will depend on the development of agents with suitable pharmacokinetic and safety profiles. The existence of TLR4 inhibitors in development for other conditions may offer repurposing opportunities [13]. Finally, these findings underscore the potential of indirect treatment strategies for gout that focus not only on urate-lowering but also on mitigating the pro-inflammatory milieu that precipitates acute attacks. A combination approach targeting both hyperuricemia and underlying metabolic inflammation, through weight loss coupled with specific immunomodulation, may be particularly relevant for patients with obesity [104,105].

### Clinical implications

Several clinical implications emerge from these findings. First, they reinforce weight management as a cornerstone of gout prevention and provide a genetic rationale for pharmacologically targeting downstream inflammatory consequences when weight loss is insufficient. Second, the identification of TLR4 as a genetically supported candidate mediator suggests that existing TLR4-directed compounds may warrant evaluation for gout, pending appropriate safety and efficacy studies. Third, the broader pleiotropy of TLR4 highlights the need for careful therapeutic window assessment, balancing anti-inflammatory efficacy against potential immunosuppressive risks [106,107]. The mixed track record of anti-inflammatory strategies in metabolic diseases – including the disappointing results of interluekin-1β blockade in large cardiovascular outcome trials despite strong mechanistic rationale – underscores the caution required when translating immunomodulatory targets from genetic evidence to clinical application [108]. Fourth, as the dual burden of obesity and gout rises rapidly in low- and middle-income countries – particularly in Asia, Latin America, and the Pacific – low-cost, generically available interventions targeting metabolic inflammation may offer practical advantages over costly biologic therapies. However, any translational application of these findings must account for ancestry-specific differences in genetic architecture and local gout epidemiology. Finally, the colocalisation of HMGCR provides genetic support for the dual urate-lowering and anti-inflammatory benefits of statins in gout patients with comorbid cardiometabolic disease.

### Future research directions

Several priorities emerge for future investigation. First, replication in non-European ancestries – including East Asian, South Asian, African, and Pacific populations – is essential to establish cross-population generalisability of TLR4 as a mediator. Such studies should examine whether TLR4-related genetic associations replicate, given known differences in allele frequencies and LD structure, and whether population-specific variants in urate transporters, such as ABCG2 and SLC2A9, modify the BMI–TLR4–gout relationship. Multi-ancestry GWAS and trans-ethnic fine-mapping will be particularly valuable for distinguishing shared from ancestry-specific causal variants. Second, tissue-specific TLR4 perturbation models are needed to distinguish direct TLR4-mediated effects from alternative pathways or intermediate phenotypes. Third, proteome-wide MR designs and plasma proteomic profiling could clarify whether circulating TLR4 levels or downstream signalling intermediates mediate the BMI–gout association. Fourth, combining weight loss interventions with TLR4-modulating strategies warrants investigation in preclinical gout models and clinical trials. Finally, systematic drug-target MR screening in independent gout cohorts is needed to validate the candidate mediators identified here and to discover additional targets.

### Limitations

This study has limitations inherent to its design and data sources. The observational findings are derived from a cross-sectional cohort, which precludes the assessment of temporality and definitive causal inference between the BMI and gout, despite statistical adjustments for key confounders [109]. More importantly, genetic analyses primarily utilised summary statistics from GWAS of European ancestry. While this enhances statistical power and leverages well-characterised cohorts, the generalisability of the identified causal effects and candidate mediators – including TLR4 – to non-European populations cannot be assumed [46]. Allele frequencies, linkage disequilibrium patterns, and genetic architecture differ substantially across ancestries, and TLR4-related associations may not replicate in populations of Asian, African, or Pacific ancestry. Until these findings are validated in diverse populations, their translational implications for precision medicine remain provisional.

Further limitations pertain to the methodological assumptions of the analytical frameworks. The MR approach relies on core assumptions that are not fully testable. Although multiple sensitivity analyses suggested minimal influence of horizontal pleiotropy, the possibility of residual bias from genetic variants that influence the outcome through pathways independent of BMI/druggable genes cannot be completely excluded [110]. Additionally, the druggable gene MR screen identified 92 genes with nominally significant effects on gout, of which only a small subset survived subsequent validation stages. This attrition may partly reflect true biological mediation but could also result from differential statistical power across loci, gene-specific linkage disequilibrium patterns affecting instrument strength, or horizontal pleiotropy undetected by sensitivity analyses. Such limitations are inherent to drug-target MR screening designs and warrant cautious interpretation of the full screening results.

The integrated computational evidence, while robust, generates a mechanistic hypothesis that requires direct validation. The specific biological pathways through which TLR4 mediates the effect of adiposity on gout pathogenesis necessitate confirmation through *in vivo* experiments in relevant model systems.

## CONCLUSIONS

In summary, this integrative study identifies TLR4 as a promising genetically supported candidate mediator linking obesity and gout, based on convergent computational prioritisation from observational, genetic, and systems-level analyses. These findings warrant further functional and translational investigation.

## Additional material


Online Supplementary Document

## Data Availability

**Data availability:** The data sets that were obtained in this study can be made available by the corresponding author upon reasonable request.
