# Supplementary material for: Genome-wide association study identifies toll-like receptor four protein-mediated metabolic remodelling affecting gout pathogenesis
Source: J Glob Health. 2026 Jul 31;16:04268. doi: 10.7189/jogh.16.04268 (PMC13424746; doi:10.7189/jogh.16.04268)

Supplement to: Li Y, Yi Z, Dong X, Sun R, Gao L, Zheng Y, Liu H. Genome-wide association study identifies toll-like receptor four protein-mediated metabolic remodelling affecting gout pathogenesis. J Glob Health. 2026;16:04268.

Online Supplementary Document

1. Checklist S1: Adherence to the Journal of Global Health's Guidelines for Reporting Analyses of Big Data Repositories Open to the Public (GRABDROP) guidelines (**Page 4-10**).

2. Checklist S2: STROBE Statement—Checklist of items that should be included in reports of *cross-sectional studies* (**Page 11-14**).

3. Checklist S3: Reproducibility (**Page 15**).

4. Table S1: Annotations of NHANES data variables from 2015 to 2018 (**Page 16-17**).

5. Table S2: Association between BMI quartiles and gout in NHANES analysis (**Page 18-19**).

6. Table S3: Subgroup analysis of the association between BMI and gout in NHANES participants (**Page 20-21**).

7. Table S4: The results of MR analysis of exposure factors and outcome factors (Inverse variance weighted *P*-value < 0.05) (**Page 22-26**).

8. Table S5: Heterogeneity analysis of exposure factors and outcome factors after MR analysis (**Page 27**).

9. Table S6: MR-PRESSO test and MR-Egger pleiotropy analysis after MR analysis of exposure and outcome factors (**Page 28**).

10. Table S7: Complete list of the SNPs used in the MR analysis of BMI and gout across multiple datasets (Inverse variance weighted *P*-value < 0.05) (**Page 29-32**).

11. Table S8: Complete MR results for BMI and Gout with druggable gene targets (Inverse variance weighted *P*-value < 0.05) (**Page 33-38**).

12. Table S9: MR analysis of genetically predicted druggable gene targets on other types of gout risk (Inverse variance weighted *P*-value < 0.05) (**Page 39-42**).

13. Table S10: Colocalization analysis of genetically predicted BMI with Gout and other types of Gout for druggable gene targets (PP.H4 > 0.75) (**Page 43**).

14. Figure S1: The workflow diagram of this study (**Page 44**).

15. Figure S2: DAG depicting the hypothesized causal framework and mediation pathways. Path a (BMI to gout): direct causal effect of adiposity on gout risk, estimated by two-sample Mendelian

randomization (MR). Path b (gene to BMI): effect of genetically proxied perturbation of a druggable gene on BMI. Path c (gene to gout): effect of the same gene on gout risk. Evidence for both paths b and c is consistent with a candidate mediating role of the gene product. Path c' (gene to gout, adjusted for BMI): BMI-independent effect of the gene on gout, estimated by multivariable MR. Dashed arrows denote potential confounding pathways (age, gender, race, etc.) that may influence both BMI and gout (**Page 45**).

16. Figure S3: Dose-response relationships between BMI and the risk of gout across study cycles and population subgroups in the NHANES cohort (**Page 46**).

17. Figure S4: Subgroup analysis of the association between continuous BMI and gout risk in the NHANES cohort (**Page 47**).

18. Figure S5: MR diagnostics for three key BMI-gout analyses (**Page 48**).

19. Figure S6: Sensitivity analyses and consistency of MR estimates for BMI and gout (**Page 49**).

20. Figure S7: Bayesian colocalization analysis identifies shared genetic loci between BMI and gout (**Page 50**).

21. Figure S8: PPI network and functional enrichment analysis of druggable gene targets (**Page 51**).

22. Figure S9: MVMR analysis of TLR4 (**Page 52**).

23. Figure S10: PheWAS analysis of Manhattan plot (**Page 53**).

Checklist S1. Adherence to the Journal of Global Health's Guidelines for Reporting Analyses of Big Data Repositories Open to the Public (GRABDROP) guidelines.

**JoGH guideline items**
**1. Please list all papers published by each co-author in previous three years that were based on secondary analysis of a big data repository.**
**Yutong Li:**<br>[1] **Li Y,** Dong X, Sun R, et al. Genomic and AI-driven discovery in chronic prostatitis: Causal role of ITPR3 and therapeutic repurposing of raloxifene. *Eur J Pharmacol*. 2026;1015:178598.<br>[2] Jiang S, **Li Y**, Lin X, Zou K, Du J, Li Q. Repurposing piroxicam as an SLC7A5 antagonist to ameliorate prothrombotic state in Diane-35-treated polycystic ovary syndrome patients. *Comput Biol Med*. 2025;198(Pt A):111187.<br>[3] **Li Y**, Lin X, Zou K, et al. Blood biochemical landscape and new insights into clinical decision-making for polycystic ovary syndrome in Chinese women: a prospective cohort study. *Front Endocrinol* (Lausanne). 2025;16:1534733.<br>[4] **Li Y**, Miao J, Liu C, et al. Kushenol O Regulates GALNT7/NF-κB axis-Mediated Macrophage M2 Polarization and Efferocytosis in Papillary Thyroid Carcinoma. *Phytomedicine*. 2025;138:156373.<br>[5] **Li Y**, Song X, Huang Y, Zhou S, Zhong L. Genetic associations of plasma metabolites with immune cells in hyperthyroidism revealed by Mendelian randomization and GWAS-sc-eQTLs xQTLbiolinks analysis. *Sci Rep*. 2025;15(1):1377.<br>[6] Tang Q, **Li Y**, Liu K, et al. Development and validation of a risk prediction model for distant metastasis in muscle-invasive bladder cancer: a retrospective study integrating SEER data with external validation cohort and biomarker analysis. *Front Oncol*. 2025;15:1607173.<br>**Zhaofeng Yi:**<br>None.

Xuan Dong:

[1] Li Y, **Dong X**, Sun R, et al. Genomic and AI-driven discovery in chronic prostatitis: Causal role of ITPR3 and therapeutic repurposing of raloxifene. *Eur J Pharmacol*. 2026;1015:178598.

Ruixu Sun:

[1] Li Y, Dong X, **Sun R**, et al. Genomic and AI-driven discovery in chronic prostatitis: Causal role of ITPR3 and therapeutic repurposing of raloxifene. *Eur J Pharmacol*. 2026;1015:178598.

Liangmeng Gao:

[1] Tang Q, Li Y, Liu K, Huang G, **Gao L**, et al. Development and validation of a risk prediction model for distant metastasis in muscle-invasive bladder cancer: a retrospective study integrating SEER data with external validation cohort and biomarker analysis. *Front Oncol*. 2025;15:1607173.

[2] Li Y, Dong X, Sun R, **Gao L**, et al. Genomic and AI-driven discovery in chronic prostatitis: Causal role of ITPR3 and therapeutic repurposing of raloxifene. *Eur J Pharmacol*. 2026;1015:178598.

Yuanning Zheng:

None.

Hongwei Liu:

[1] Tang Q, Li Y, Liu K, Huang G, Gao L, Tang Y, **Liu H**. Development and validation of a risk prediction model for distant metastasis in muscle-invasive bladder cancer: a retrospective study integrating SEER data with external validation cohort and biomarker analysis. *Front Oncol*. 2025;15:1607173.

[2] [2] Li Y, Dong X, Sun R, Gao L, Song X, Huang Z, Zuo L, **Liu H**. Genomic and AI-driven discovery in chronic prostatitis: Causal role of ITPR3 and therapeutic repurposing of raloxifene. *Eur J Pharmacol*. 2026;1015:178598.

2. Please explain the key elements of your study design and the use of the available datasets that make your study an original scientific contribution.

Our study's originality lies in the integration of multiple analytical layers and the systematic prioritization of druggable gene mediators, rather than in the use of novel datasets.

[1] Key elements of study design:

Multi-stage framework: We implemented a three-stage analytical hierarchy—(i) observational confirmation in NHANES; (ii) two-sample Mendelian randomization (MR) to establish causality; (iii) systematic screening of 113 druggable gene loci with multi-layered validation including Bayesian colocalization, protein–protein interaction network analysis, linkage disequilibrium score regression, and multivariable MR.
Drug-target MR screening: Rather than testing a single candidate gene, we performed a hypothesis-generating screen across 113 druggable genes, followed by stringent filtering to identify TLR4 as a genetically supported mediator.
Convergent evidence: The prioritization of TLR4 was supported by convergent evidence from five orthogonal approaches—MR, colocalization (PP.H4 > 0.75), SMR with HEIDI testing, network centrality, and MVMR demonstrating BMI-independent effects.
[2] Use of available datasets:
NHANES (2015–2018): Provided observational dose–response characterisation of the BMI–gout association in a nationally representative, multi-ancestry sample (n = 9,700), establishing the epidemiological foundation for subsequent genetic analyses.
OpenGWAS/UK Biobank/GIANT: Provided BMI genetic instruments from large-scale European-ancestry GWAS meta-analyses (n = 681,275) for MR analyses.
FinnGen (GOUT_STRICT, M13_GOUT, and four clinical subtypes): Served as the primary outcome dataset, enabling both primary gout analyses and subtype-stratified consistency assessments.
eQTLGen / GTEx (version 8): Provided cis-eQTL data for SMR analyses to integrate gene expression with GWAS findings.
STRING, Cytoscape, MCODE: Used for protein–protein interaction network analysis to assess functional connectivity.
[3] Original scientific contribution:
To our knowledge, this is the first study to systematically apply a drug-target MR screening framework to identify mediators of the BMI–gout causal pathway. While prior MR studies have confirmed the causal effect of BMI on gout, none have systematically screened druggable genes and validated candidates through colocalization, network analysis, genetic correlation, and

BMI-independence testing. Our convergent evidence identifies TLR4 as a genetically supported candidate mediator linking obesity-driven metabolic dysregulation to gout-associated inflammation—a finding with potential therapeutic implications.

3. Please list all publications that addressed similar research questions in the same dataset and indicate where you cited them in your paper.

To the best of our knowledge, the following published studies have addressed similar research questions—specifically, the causal relationship between BMI and gout—using overlapping or identical datasets to those employed in our study. We have cited these studies in the appropriate sections of our manuscript, as indicated below.

[1] Karlsson T, Hadizadeh F, Rask-Andersen M, Johansson Å, Ek WE. Body Mass Index and the Risk of Rheumatic Disease: Linear and Nonlinear Mendelian Randomization Analyses. *Arthritis Rheumatol*. 2023;75:2027-35.

[Estimated the causal effect of BMI on risk of 5 rheumatic diseases including gout using linear and nonlinear MR; identified sex-specific effects.]

Cited in Introduction and Discussion [11]

[2] Adams CD, Boutwell BB. Using multiple Mendelian randomization approaches and genetic correlations to understand obesity, urate, and gout. *Sci Rep*. 2021;11:17799.

[Used multiple MR approaches and genetic correlations to understand the relationships between obesity, urate, and gout.]

Cited in Discussion [70]

[3] He C, Zhang M, Li J, Wang Y, Chen L, Qi B, et al. Novel insights into the consequences of obesity: a phenotype-wide Mendelian randomization study. *Eur J Hum Genet*. 2022;30:540-6.

[Phenome-wide MR evaluating health consequences of obesity; confirmed gout as a consequence of obesity.]

Cited in Discussion [71]

[4] Topless RKG, et al. The comparative effect of exposure to various risk factors on the risk of hyperuricaemia: diet has a weak causal effect. *Arthritis Res Ther*. 2021;23.

[Compared attributable fractions of genetic, dietary, BMI, and other risk factors for hyperuricaemia; used two-sample MR.]

Cited in Introduction [9]

[5] Yang T, Bi S, Zhang X, Yin M, Feng S, Li H. The Impact of Different Intensities of Physical Activity on Serum Urate and Gout: A Mendelian Randomization Study. *Metabolites*. 2024;14:66.

[Explored causal effects of different physical activity intensities on serum urate and gout using two-sample MR.]

Not cited in our paper (focuses on physical activity rather than BMI)

The literature marked as Not cited in the table above has research questions that differ from our paper, therefore they were not cited in the main text. According to the spirit of the GRADROP guidelines, we truthfully list all known relevant studies, whether or not they have been cited in the paper.

In addition, the following studies used the NHANES database to explore the association between BMI and gout/hyperuricemia, but did not use MR methods (mainly cross-sectional observational studies), which differs from the core method of our paper (MR causal inference):

Li et al. Association between non-high-density lipoprotein cholesterol to high-density lipoprotein cholesterol ratio (NHHR) and gout in US adults: a cross-sectional study of the mediating role of BMI. *Diabetol Metab Syndr*. 2025; 17:223.

Relationship between gout, hyperuricemia, and obesity—does central obesity play a significant role? —a study based on the NHANES database. *Diabetol Metab Syndr*. 2024; 16:24.

Although the observational study mentioned above used the same dataset (NHANES), the study design and analytical framework differ fundamentally from our MR study. We have covered this background knowledge in the Introduction of our paper by citing relevant literature (such as Dehlin et al., 2020 and Choi et al., 2005).

4. Please explain how you addressed multiple testing through an appropriately rigorous statistical threshold and indicate this in the methods section.

We addressed multiple testing through a multi-layered statistical strategy that applies different thresholds appropriate to each analytical stage, and all thresholds are clearly specified in the Methods section.

In the primary MR screening of 113 druggable gene loci, we controlled the false discovery rate

using the Benjamini-Hochberg procedure, with an FDR < 0.20 considered suggestive and *FDR* < 0.05 considered statistically significant; this is stated in the "MR analyses" subsection of Methods.

For the two-sample MR establishing the causal effect of BMI on gout, we applied the conventional genome-wide significance threshold of $P < 5 \times 10^{-8}$ for instrument selection, with sensitivity analyses (MR-Egger, weighted median, MR-PRESSO) and Cochran's *Q* test for heterogeneity; this is detailed in the "MR analyses" subsection. For the druggable gene loci where no SNP reached genome-wide significance, we applied a more liberal instrument-wide threshold of $P < 1 \times 10^{-5}$, justified by the limited number of cis-SNPs within the ± 10 kb window, established precedent in drug-target MR, and the fact that these candidates were subjected to stringent subsequent filtering including colocalization (PP.H4 > 0.75), HEIDI testing ($P > 0.05$), and *F*-statistic > 10; this is also described in the "Exposure data sources and instrument selection" and "MR analyses" subsections of Methods.

For Bayesian colocalization, we prespecified PP.H4 > 0.75 as strong evidence for shared causal variants, as stated in the "Bayesian colocalization analysis" subsection. For the SMR analyses, we applied the HEIDI test with $P > 0.05$ to rule out false-positive associations due to linkage disequilibrium, as described in the "SMR analysis" subsection.

For genome-wide genetic correlation using LDSC, statistical significance was defined as $P < 0.05$, as stated in the "Genetic correlation analysis using linkage disequilibrium score regression (LDSC)" subsection. Importantly, our primary finding—TLR4 as a candidate mediator—was not based on a single statistical threshold; rather, it emerged from the convergence of multiple independent lines of evidence (MR, colocalization, SMR, PPI network centrality, LDSC, and MVMR), providing robustness beyond any individual *P*-value threshold. This multi-layered validation approach, with all thresholds explicitly reported in the Methods section, mitigates the risk of false-positive findings inherent to large-scale screening analyses.

5. Please declare to what extent have AI chatbots been used in developing your paper and to which parts of the paper did they contribute.

No AI chatbots was used during the manuscript preparation.

AI – artificial intelligence, JoGH – Journal of Global Health, GRABDROP – Guidelines for

Reporting Analyses of Big Data Repositories Open to the Public, NHANES – National Health and Nutrition Examination Survey, UK Biobank – United Kingdom Biobank, GIANT – Genetic Investigation of ANthropometric Traits, FinnGen – Finnish Genome Study, GTEx – Genotype-Tissue Expression, eQTLGen – Expression Quantitative Trait Loci Consortium, STRING – Search Tool for the Retrieval of Interacting Genes/Proteins, MCODE – Molecular Complex Detection, IEU – MRC Integrative Epidemiology Unit, MR – Mendelian randomization, SMR – Summary-data-based Mendelian randomization, MVMR – Multivariable Mendelian randomization, LDSC – Linkage disequilibrium score regression, PPI – Protein–protein interaction, GWAS – Genome-wide association study, SNP – Single-nucleotide polymorphism, eQTL – Expression quantitative trait locus, cis-eQTL – cis-expression quantitative trait locus, HEIDI – HEterogeneity In Dependent Instruments, IVW – Inverse-variance weighted, MR-PRESSO – MR pleiotropy RESidual sum and outlier, FDR – False discovery rate, PP – Posterior probability, PP.H4 – Posterior probability of hypothesis 4 (shared causal variant), LD – Linkage disequilibrium, OR – Odds ratio, CI – Confidence interval, SE – Standard error, KEGG – Kyoto Encyclopedia of Genes and Genomes, GO – Gene ontology, PheWAS – Phenome-wide association study, DAG – Directed acyclic graph, RCS – Restricted cubic spline, TLR4 – Toll-like receptor 4, BMI – Body mass index, NLRP3 – NOD-like receptor thermal protein domain associated protein 3, IL-1β – Interleukin-1 beta, IL1A – Interleukin-1 alpha, NF-κB – Nuclear factor kappa-light-chain-enhancer of activated B cells, JAK2 – Janus kinase 2, NFKBIA – NFKB inhibitor alpha, CCR6 – C-C chemokine receptor type 6, FCGR3B – Fc gamma receptor IIIb, CD33 – Cluster of differentiation 33, HMGCR – 3-hydroxy-3-methylglutaryl-CoA reductase, MAPK3 – Mitogen-activated protein kinase 3, SEMA6A – Semaphorin 6A, INVS – Inversin, GATM – Glycine amidinotransferase, HOXB4 – Homeobox B4, ABCG2 – ATP binding cassette subfamily G member 2, SLC2A9 – Solute carrier family 2 member 9, SLC7A5 – Solute carrier family 7 member 5, ITPR3 – Inositol 1,4,5-trisphosphate receptor type 3, GALNT7 – Polypeptide N-acetylgalactosaminyltransferase 7.

Checklist S2. STROBE Statement—Checklist of items that should be included in reports of *cross-sectional studies*.

	Item No	Recommendation	Page No
**Title and abstract**	1	(*a*) Indicate the study's design with a commonly used term in the title or the abstract	1-2
		(*b*) Provide in the abstract an informative and balanced summary of what was done and what was found	2
**Introduction**			
Background/rationale	2	Explain the scientific background and rationale for the investigation being reported	2-4
Objectives	3	State specific objectives, including any prespecified hypotheses	4
**Methods**			
Study design	4	Present key elements of study design early in the paper	3
Setting	5	Describe the setting, locations, and relevant dates, including periods of recruitment, exposure, follow-up, and data collection	6
Participants	6	(*a*) Give the eligibility criteria, and the sources and methods of selection of participants	6
Variables	7	Clearly define all outcomes, exposures, predictors, potential confounders, and effect modifiers. Give diagnostic criteria, if applicable	4-7

Data sources/ measurement	8*	For each variable of interest, give sources of data and details of methods of assessment (measurement). Describe comparability of assessment methods if there is more than one group	6, 35-36
Bias	9	Describe any efforts to address potential sources of bias	6-7
Study size	10	Explain how the study size was arrived at	6
Quantitative variables	11	Explain how quantitative variables were handled in the analyses. If applicable, describe which groupings were chosen and why	6-7, 36-37
Statistical methods	12	(*a*) Describe all statistical methods, including those used to control for confounding	6-7, 9-10
		(*b*) Describe any methods used to examine subgroups and interactions	6-7
		(*c*) Explain how missing data were addressed	6
		(*d*) If applicable, describe analytical methods taking account of sampling strategy	6-7
		(*e*) Describe any sensitivity analyses	6-9
**Results**			
Participants	13*	(a) Report numbers of individuals at each stage of study—eg numbers potentially eligible, examined for eligibility, confirmed eligible, included in the study, completing follow-up, and analysed	6
		(b) Give reasons for non-participation at each stage	6
		(c) Consider use of a flow diagram	4
Descriptive data	14*	(a) Give characteristics of study participants (eg demographic, clinical, social) and information on exposures and potential confounders	10-11, 36-37
		(b) Indicate number of participants with missing data	6

		for each variable of interest	
Outcome data	15*	Report numbers of outcome events or summary measures	10-11, 36-37
Main results	16	(*a*) Give unadjusted estimates and, if applicable, confounder-adjusted estimates and their precision (eg, 95% confidence interval). Make clear which confounders were adjusted for and why they were included	6-7, 37-38
		(*b*) Report category boundaries when continuous variables were categorized	7, 37
		(*c*) If relevant, consider translating estimates of relative risk into absolute risk for a meaningful time period	N/A
Other analyses	17	Report other analyses done—eg analyses of subgroups and interactions, and sensitivity analyses	12-13, 15-16
**Discussion**			
Key results	18	Summarise key results with reference to study objectives	16, 19-20
Limitations	19	Discuss limitations of the study, taking into account sources of potential bias or imprecision. Discuss both direction and magnitude of any potential bias	20
Interpretation	20	Give a cautious overall interpretation of results considering objectives, limitations, multiplicity of analyses, results from similar studies, and other relevant evidence	16-19
Generalisability	21	Discuss the generalisability (external validity) of the study results	16-19
**Other information**			

Funding	22	Give the source of funding and the role of the funders for the present study and, if applicable, for the original study on which the present article is based	21

* Give information separately for exposed and unexposed groups.

Note: An Explanation and Elaboration article discusses each checklist item and gives methodological background and published examples of transparent reporting. The STROBE checklist is best used in conjunction with this article (freely available on the Web sites of PLoS Medicine at http://www.plosmedicine.org/, Annals of Internal Medicine at http://www.annals.org/, and Epidemiology at http://www.epidem.com/). Information on the STROBE Initiative is available at www.strobe-statement.org.

STROBE – Strengthening the Reporting of Observational Studies in Epidemiology, N/A – not applicable, e.g. – exempli gratia (for example), etc. – et cetera (and so on), OR – odds ratio, CI – confidence interval.

Checklist S3. Reproducibility.

Software and key parameters. Two-sample MR analyses were conducted using the TwoSampleMR package (vaersion 0.5.7). MR-Egger regression, weighted median, and IVW methods were implemented with default parameters. Heterogeneity was assessed using Cochran's Q statistic ($P <$ 0.05 considered significant heterogeneity). Horizontal pleiotropy was evaluated using the MR-Egger intercept test and the MR-PRESSO global test (version 1.0). Colocalization was performed using the coloc package (version 5.2.1) with default priors ($p_1 = 1\times10^{-4}$, $p_2 = 1\times10^{-4}$, $p_{12} = 1\times10^{-5}$). SMR analysis used SMR software (version 1.3.1) with default parameters. HEIDI testing was performed with a significance threshold of $P > 0.05$ for non-rejection of the shared variant hypothesis. LDSC was conducted using ldsc (version 1.0.1) with both unconstrained and constrained intercept models. PPI networks were constructed using STRING (version 12.0) with a high-confidence interaction score threshold of > 0.9, and visualized in Cytoscape (version 3.7.0). MCODE clustering used default parameters. Functional enrichment used the clusterProfiler package (version 4.8.1) with Benjamini-Hochberg FDR correction. MVMR was performed using the MVMR package (version 0.4) in R.

MR – Mendelian randomization, IVW – inverse-variance weighted, MR-PRESSO – MR pleiotropy RESidual sum and outlier, SMR – summary-data-based Mendelian randomization, HEIDI – HEterogeneity In Dependent Instruments, LDSC – linkage disequilibrium score regression, PPI – protein–protein interaction, MCODE – molecular complex detection, MVMR – multivariable Mendelian randomization, FDR – false discovery rate, CI – confidence interval, STRING – Search Tool for the Retrieval of Interacting Genes/Proteins, e.g. – exempli gratia (for example).

Table S1. Annotations of NHANES data variables from 2015 to 2018.

File	Variable	Label	Type	Code	Value
DEMO_I	RIAGENDR	Gender	discrete	1	Male
DEMO_I	RIAGENDR	Gender	discrete	2	Female
DEMO_I	RIDAGEYR	Age	continuous	/	/
DEMO_I	RIDRETH1	Race	discrete	1	Mexican American
DEMO_I	RIDRETH1	Race	discrete	2	Other Hispanic
DEMO_I	RIDRETH1	Race	discrete	3	Non-Hispanic White
DEMO_I	RIDRETH1	Race	discrete	4	Non-Hispanic Black
DEMO_I	RIDRETH1	Race	discrete	5	Other Race - Including Multi-Racial
DEMO_I	DMDEDUC2	Education	discrete	1	Less than 9th grade
DEMO_I	DMDEDUC2	Education	discrete	2	9-11th grade (Includes 12th grade with no diploma)
DEMO_I	DMDEDUC2	Education	discrete	3	High school graduate/GED/equivalent
DEMO_I	DMDEDUC2	Education	discrete	4	Some college/AA degree
DEMO_I	DMDEDUC2	Education	discrete	5	College graduate/above
DEMO_I	DMDEDUC2	Education	discrete	7	Refused
DEMO_I	DMDEDUC2	Education	discrete	9	Don't Know

DEMO_I	WTINT2YR	WTINT2YR	continuous	/	/
DEMO_I	WTMEC2YR	WTMEC2YR	continuous	/	/
DEMO_I	SDMVPSU	SDMVPSU	continuous	/	/
DEMO_I	SDMVSTRA	SDMVSTRA	continuous	/	/
BMX_I	BMXBMI	exposure.BMI	continuous	/	/
MCQ_I	MCQ160n	outcome.Gout	discrete	1	Yes
MCQ_I	MCQ160n	outcome.Gout	discrete	2	No
MCQ_I	MCQ160n	outcome.Gout	discrete	7	Refused
MCQ_I	MCQ160n	outcome.Gout	discrete	9	Don't know
ALQ_I	ALQ101	Alcohol	discrete	1	Yes
ALQ_I	ALQ101	Alcohol	discrete	2	No
ALQ_I	ALQ101	Alcohol	discrete	7	Refused
ALQ_I	ALQ101	Alcohol	discrete	9	Don't know
SMQ_I	SMQ020	Smoke	discrete	1	Yes
SMQ_I	SMQ020	Smoke	discrete	2	No
SMQ_I	SMQ020	Smoke	discrete	7	Refused
SMQ_I	SMQ020	Smoke	discrete	9	Don't know
DR1TOT_I	DR1TFA	Folic	continuous	/	/

DEMO_I – demographics file (2015–2016 cycle), BMX_I – body measures file (2015–2016 cycle), MCQ_I – medical conditions file (2015–2016 cycle), ALQ_I – alcohol use file (2015–2016 cycle), SMQ_I – smoking questionnaire file (2015–2016 cycle), DR1TOT_I – dietary recall (total nutrients) file (2015–2016 cycle), NHANES – National Health and Nutrition Examination Survey, RIAGENDR – respondent id – gender, RIDAGEYR – respondent id – age in years, RIDRETH1 – respondent id – race/ethnicity (1st version), DMDEDUC2 – demographic – education level (2nd version), WTINT2YR – interview weight – 2-year, WTMEC2YR – examination weight – 2-year, SDMVPSU – masked variance – primary sampling unit, SDMVSTRA – masked variance – strata, BMXBMI – body measures – body mass index, MCQ160n – medical conditions – question 160 (gout) – number, ALQ101 – alcohol use – question 101, SMQ020 – smoking – question 020, DR1TFA – dietary recall – total folic acid, GED – general educational development, AA – associate of arts, BMI – body mass index.

Table S2. Association between BMI quartiles and gout in NHANES analysis.

Variable	Model 1 [a]		Model 2 [b]		Model 3 [c]	
	OR (95% CI)	*P*-value	OR (95% CI)	*P*-value	OR (95% CI)	*P*-value
**Exposure Quartile (Ref: Q1)**						
Q2	1.86 (1.29–2.69)	0.003	1.40 (0.96–2.02)	0.095	1.39 (0.96–2.00)	0.101
Q3	2.24 (1.40–3.58)	0.002	1.79 (1.09–2.94)	0.036	1.75 (1.06–2.87)	0.045
Q4	2.92 (1.99–4.29)	< 0.001	2.89 (1.93–4.33)	< 0.001	2.83 (1.87–4.26)	< 0.001
**Demographic Factors**						
Gender (Ref: Female)						
Male	/	/	2.71 (2.05–3.57)	< 0.001	2.47 (1.83–3.32)	< 0.001
Age (per year)	/	/	1.06 (1.05–1.07)	< 0.001	1.06 (1.05–1.07)	< 0.001
Race/Ethnicity (Ref: Mexican American)						
Non-Hispanic Black	/	/	1.64 (1.05–2.56)	0.045	1.61 (1.03–2.54)	0.057
Non-Hispanic White	/	/	1.22 (0.80–1.86)	0.370	1.19 (0.78–1.80)	0.440
Other Hispanic	/	/	1.13 (0.66–1.93)	0.660	1.14 (0.66–1.94)	0.650
Other/Multiracial	/	/	1.96 (1.23–	0.011	2.02 (1.28–	0.009

			3.12)		3.19)	
**Education (Ref: < 9th Grade)**						
9-11th grade	/	/	1.21 (0.74–1.99)	0.460	1.09 (0.64–1.84)	0.760
High school graduate	/	/	1.21 (0.77–1.88)	0.420	1.10 (0.69–1.73)	0.700
Some college/AA degree	/	/	1.48 (0.95–2.31)	0.100	1.37 (0.88–2.14)	0.190
College graduate/above	/	/	1.01 (0.55–1.86)	0.970	0.97 (0.54–1.73)	0.920
**Lifestyle Factors**						
Alcohol use (Ref: No)						
Yes	/	/	/	/	1.59 (1.20–2.10)	0.006
Folic acid	/	/	/	/	1.00 (1.00–1.00) [&]	0.068
Smoking (Ref: No)						
Yes	/	/	/	/	1.37 (0.98–1.93)	0.087

[a] Model 1: Unadjusted analysis.

[b] Model 2: Adjusted for all demographic factors listed (gender, age, race/ethnicity, and education level).

[c] Model 3: Adjusted for all variables in Model 2 plus lifestyle factors (alcohol use, folic acid intake, and smoking status).

[&] For folic acid (continuous), the exact OR is 0.9992 (95% CI: 0.9983–0.999994). The value is displayed as 1.00 due to rounding.

BMI – body mass index, NHANES – national health and nutrition examination survey.

Table S3. Subgroup analysis of the association between BMI and gout in NHANES participants.

Subgroup	Group	β (95% CI)	*P*-value	*P*-interaction
Gender	/	/	/	0.600
	Female	0.045 (0.027–0.063)	< 0.001	
	Male	0.050 (0.035–0.065)	< 0.001	
Age (years)	/	/	/	0.220
	≤ 65	0.042 (0.023–0.061)	< 0.001	
	> 65	0.065 (0.038–0.092)	< 0.001	
Race/Ethnicity	/	/	/	0.110
	Mexican American	0.055 (0.012–0.097)	0.020	
	Non-Hispanic Black	0.012 (−0.006–0.030)	0.190	
	Non-Hispanic White	0.050 (0.033–0.066)	< 0.001	
	Other Hispanic	0.045 (−0.006–0.097)	0.100	
	Other/Multiracial	0.047 (0.014–0.079)	0.010	
Education	/	/	/	0.020
	< 9th grade	0.055 (0.012–0.097)	0.020	
	9–11th grade	0.041 (0.014–0.068)	0.010	
	High school graduate	0.009 (−0.011–0.030)	0.390	
	Some college/AA degree	0.050 (0.032–0.068)	< 0.001	
	College graduate/above	0.056 (0.028–0.084)	<0.001	

Alcohol use	/	/	/	0.400
	No	0.056 (0.031–0.082)	< 0.001	
	Yes	0.041 (0.026–0.056)	< 0.001	
Folic acid quartile	/	/	/	0.120
	Q1 (lowest)	0.064 (0.040–0.087)	< 0.001	
	Q2	0.023 (−0.002–0.048)	0.080	
	Q3	0.027 (−0.010–0.064)	0.160	
	Q4 (highest)	0.056 (0.033–0.079)	< 0.001	
Smoking status	/	/	/	0.760
	No	0.044 (0.023–0.066)	< 0.001	
	Yes	0.040 (0.024–0.056)	< 0.001	

[&] The estimates (β) represent the change in the outcome per unit increase in exposure within each subgroup, derived from linear regression models.

NHANES – national health and nutrition examination survey, BMI – body mass index.

Table S4. The results of MR analysis of exposure factors and outcome factors (Inverse variance weighted *P*-value < 0.05).

Exposure	Outcome	SNPs	β (95% CI)	OR (95% CI)	*P*-value
bbj-a-3	ieu-a-1054	16	0.466 (0.127 to 0.805)	1.593 (1.135 to 2.236)	0.007
ebi-a-GCST004904	ebi-a-GCST001790	40	0.390 (0.073 to 0.707)	1.477 (1.076 to 2.028)	0.016
bbj-a-1	ukb-b-12765	20	0.002 (0.000 to 0.003)	1.002 (1.000 to 1.003)	0.037
bbj-a-1	ukb-b-13251	51	0.003 (0.001 to 0.006)	1.003 (1.001 to 1.006)	0.013
bbj-a-1	ukb-a-107	56	0.003 (0.000 to 0.006)	1.003 (1.000 to 1.006)	0.036
bbj-a-1	ebi-a-GCST001790	37	0.339 (0.032 to 0.646)	1.404 (1.033 to 1.908)	0.030
bbj-a-1	ieu-a-1054	37	0.339 (0.032 to 0.646)	1.404 (1.033 to 1.908)	0.030
bbj-a-2	ukb-b-13251	20	0.004 (0.001 to 0.007)	1.004 (1.001 to 1.007)	0.022
bbj-a-2	ukb-a-107	23	0.004 (0.000 to 0.007)	1.004 (1.000 to 1.007)	0.036
ebi-a-GCST002783	ukb-b-12765	36	0.002 (0.000 to 0.003)	1.002 (1.000 to 1.003)	0.012
ebi-a-GCST002783	ukb-b-13251	66	0.004 (0.002 to 0.007)	1.004 (1.002 to 1.007)	0.001

ebi-a-GCST002783	ukb-a-107	67	0.004 (0.001 to 0.007)	1.004 (1.001 to 1.007)	0.008
ebi-a-GCST002783	ebi-a-GCST001790	73	0.704 (0.405 to 1.004)	2.023 (1.499 to 2.729)	< 0.0001
ebi-a-GCST002783	ieu-a-1054	74	0.693 (0.395 to 0.991)	1.999 (1.484 to 2.693)	< 0.0001
ebi-a-GCST002783	finn-b-M13_GOUT	65	0.421 (0.172 to 0.670)	1.523 (1.187 to 1.953)	0.001
ebi-a-GCST002783	finn-b-GOUT	68	0.426 (0.184 to 0.668)	1.531 (1.202 to 1.950)	0.001
ebi-a-GCST002783	finn-b-GOUT_STRICT	69	0.374 (0.030 to 0.718)	1.453 (1.030 to 2.050)	0.033
ebi-a-GCST002783	finn-b-GOUT_NOS	66	0.409 (0.089 to 0.729)	1.506 (1.093 to 2.074)	0.012
ebi-a-GCST004904	ukb-b-13251	52	0.003 (0.000 to 0.005)	1.003 (1.000 to 1.005)	0.036
ebi-a-GCST004904	ukb-a-107	56	0.003 (0.000 to 0.006)	1.003 (1.000 to 1.006)	0.025
ebi-a-GCST004904	finn-b-M13_GOUT	56	0.273 (0.033 to 0.514)	1.314 (1.033 to 1.671)	0.026
ebi-a-GCST90018727	ukb-b-13251	59	0.004 (0.002 to 0.007)	1.004 (1.002 to 1.007)	0.001
ebi-a-GCST90018727	ukb-a-107	63	0.004 (0.001 to 0.007)	1.004 (1.001 to 1.007)	0.007
ebi-a-GCST90018727	ebi-a-GCST001790	53	0.730 (0.350 to 1.109)	2.075 (1.420 to 3.032)	< 0.0001
ebi-a-GCST90018727	ieu-a-1054	53	0.730 (0.350 to 1.109)	2.075 (1.420 to 3.032)	< 0.0001
ieu-a-2	ukb-b-12765	37	0.002 (0.000	1.002 (1.000	0.014

			to 0.003)	to 1.003)	
ieu-a-2	ukb-b-13251	69	0.004 (0.002 to 0.007)	1.004 (1.002 to 1.007)	< 0.0001
ieu-a-2	ukb-a-107	70	0.004 (0.001 to 0.007)	1.004 (1.001 to 1.007)	0.006
ieu-a-2	ebi-a-GCST001790	72	0.679 (0.378 to 0.981)	1.972 (1.459 to 2.667)	< 0.0001
ieu-a-2	ieu-a-1054	73	0.670 (0.370 to 0.971)	1.955 (1.448 to 2.639)	< 0.0001
ieu-a-2	finn-b-M13_GOUT	68	0.423 (0.177 to 0.668)	1.526 (1.194 to 1.951)	< 0.0001
ieu-a-2	finn-b-GOUT	71	0.428 (0.190 to 0.667)	1.535 (1.209 to 1.948)	< 0.0001
ieu-a-2	finn-b-GOUT_STRICT	72	0.370 (0.031 to 0.710)	1.448 (1.031 to 2.033)	0.032
ieu-a-2	finn-b-GOUT_NOS	69	0.410 (0.095 to 0.726)	1.507 (1.099 to 2.067)	0.011
ieu-a-95	ukb-b-13251	7	0.006 (0.002 to 0.009)	1.006 (1.002 to 1.009)	0.004
ieu-a-95	ukb-a-107	7	0.005 (0.000 to 0.009)	1.005 (1.000 to 1.009)	0.035
ieu-a-95	ebi-a-GCST001790	9	0.617 (0.166 to 1.068)	1.853 (1.180 to 2.908)	0.007
ieu-a-95	ieu-a-1054	9	0.617 (0.166 to 1.068)	1.853 (1.180 to 2.908)	0.007
ieu-a-95	finn-b-M13_GOUT	9	0.562 (0.198 to 0.926)	1.754 (1.219 to 2.525)	0.002
ieu-a-95	finn-b-GOUT	9	0.441 (0.081 to 0.800)	1.554 (1.085 to 2.226)	0.016

ieu-a-785	ukb-b-13251	25	0.004 (0.001 to 0.007)	1.004 (1.001 to 1.007)	0.020
ieu-a-785	ukb-a-107	27	0.005 (0.001 to 0.008)	1.005 (1.001 to 1.008)	0.006
ieu-a-785	ebi-a-GCST001790	26	0.524 (0.145 to 0.904)	1.689 (1.156 to 2.469)	0.007
ieu-a-785	ieu-a-1054	26	0.524 (0.145 to 0.904)	1.689 (1.156 to 2.469)	0.007
ieu-a-785	finn-b-M13_GOUT	26	0.451 (0.151 to 0.750)	1.569 (1.163 to 2.117)	0.003
ieu-a-785	finn-b-GOUT	27	0.316 (0.024 to 0.609)	1.372 (1.024 to 1.838)	0.034
ieu-a-835	ukb-b-12765	30	0.002 (0.000 to 0.003)	1.002 (1.000 to 1.003)	0.024
ieu-a-835	ukb-b-13251	60	0.005 (0.002 to 0.007)	1.005 (1.002 to 1.007)	< 0.0001
ieu-a-835	ukb-a-107	60	0.004 (0.001 to 0.008)	1.004 (1.001 to 1.008)	0.006
ieu-a-835	ebi-a-GCST001790	62	0.587 (0.252 to 0.922)	1.799 (1.287 to 2.515)	< 0.0001
ieu-a-835	ieu-a-1054	63	0.577 (0.244 to 0.910)	1.781 (1.276 to 2.485)	< 0.0001
ieu-a-835	finn-b-M13_GOUT	56	0.369 (0.089 to 0.650)	1.447 (1.093 to 1.915)	0.010
ieu-a-835	finn-b-GOUT	61	0.422 (0.154 to 0.689)	1.524 (1.167 to 1.992)	0.002
ieu-a-835	finn-b-GOUT_STRICT	61	0.433 (0.052 to 0.815)	1.542 (1.053 to 2.258)	0.026
ieu-a-835	finn-b-	59	0.368 (0.013	1.445 (1.013	0.042

	GOUT_NOS		to 0.724)	to 2.062)	
ieu-a-974	ukb-b-12765	17	0.002 (0.000 to 0.003)	1.002 (1.000 to 1.003)	0.029
ieu-a-974	ukb-b-13251	30	0.004 (0.001 to 0.007)	1.004 (1.001 to 1.007)	0.004
ieu-a-974	ukb-a-107	29	0.004 (0.001 to 0.007)	1.004 (1.001 to 1.007)	0.011
ieu-a-974	ebi-a-GCST001790	34	0.676 (0.341 to 1.010)	1.965 (1.407 to 2.745)	< 0.0001
ieu-a-974	ieu-a-1054	34	0.676 (0.341 to 1.010)	1.965 (1.407 to 2.745)	< 0.0001
ieu-b-4816	ukb-b-12765	15	0.000 (0.000 to 0.001)	1.000 (1.000 to 1.001)	0.006
ieu-b-4816	ukb-b-13251	24	0.001 (0.000 to 0.002)	1.001 (1.000 to 1.002)	0.004
ieu-b-4816	ukb-a-107	28	0.001 (0.000 to 0.002)	1.001 (1.000 to 1.002)	0.004
ieu-b-4816	ebi-a-GCST001790	29	0.119 (0.042 to 0.195)	1.126 (1.043 to 1.216)	0.002
ieu-b-4816	ieu-a-1054	29	0.119 (0.042 to 0.195)	1.126 (1.043 to 1.216)	0.002
ieu-b-4816	finn-b-M13_GOUT	32	0.078 (0.015 to 0.142)	1.082 (1.015 to 1.153)	0.016
ieu-b-4816	finn-b-GOUT	32	0.062 (0.002 to 0.122)	1.064 (1.002 to 1.130)	0.044
ieu-b-4816	finn-b-GOUT_STRICT	32	0.095 (0.011 to 0.178)	1.099 (1.011 to 1.195)	0.027
ieu-b-4816	finn-b-GOUT_NOS	32	0.081 (0.004 to 0.158)	1.084 (1.004 to 1.171)	0.039

MR – mendelian randomization, OR – odd ratio, CI – confidence interval, SNP – single nucleotide

polymorphism.

Table S5. Heterogeneity analysis of exposure factors and outcome factors after MR analysis.

Exposure dataset	Method	*Q* statistic	df	*P*-value
bbj-a-3	MR Egger	6.522	14	0.952
	Inverse variance weighted	9.139	15	0.870
ebi-a-GCST004904	MR Egger	25.905	38	0.932
	Inverse variance weighted	25.926	39	0.946
ieu-a-974	MR Egger	12.513	32	0.999
	Inverse variance weighted	12.541	33	0.999

MR – mendelian randomization.

Table S6. MR-PRESSO test and MR-Egger pleiotropy analysis after MR analysis of exposure and outcome factors.

Exposure dataset	MR-PRESSO *P*-value	MR-Egger *P*-value
bbj-a-3	0.824	0.128
ebi-a-GCST004904	0.950	0.884
ieu-a-974	1.000	0.868

MR – mendelian randomization.

Table S7. Complete list of the SNPs used in the MR analysis of BMI and gout across multiple datasets (Inverse variance weighted *P*-value < 0.05).

SNP	Chr	Position	Effect Allele	*P*-value (BMI)	*P*-value (Gout)
rs1016287	2	59305625	C	5.87E-09	0.897
rs10182181	2	25150296	G	3.91E-21	0.380
rs10205578	2	51185310	G	2.04E-08	0.614
rs10208649	2	54161363	C	6.47E-11	0.666
rs1035491	5	63966889	G	2.68E-09	0.535
rs10733682	9	129460914	G	1.66E-08	0.110
rs10764373	10	18553968	T	4.59E-09	0.954
rs10807139	6	34197188	C	1.87E-12	0.626
rs10811658	9	22128600	A	3.61E-09	0.159
rs10835389	11	28676505	C	3.97E-08	0.787
rs10938397	4	45182527	G	2.98E-23	0.388
rs10968576	9	28414339	G	1.03E-11	0.568
rs11030100	11	27684517	T	2.51E-26	0.327
rs11165643	1	96924097	T	5.76E-09	0.874
rs1121980	16	53809247	A	4.87E-86	0.111
rs11602339	11	47763016	T	8.14E-10	0.900
rs11642015	16	53805207	T	4.91E-72	0.077
rs11663558	18	21133937	A	4.37E-09	0.149
rs12529728	6	50896630	G	1.76E-19	0.540
rs12597682	16	20258432	A	6.80E-11	0.783
rs13098327	3	85820181	A	1.12E-11	0.338

rs138773516	8	65186105	T	6.75E-09	0.680
rs1442493	4	100321365	A	3.02E-08	0.419
rs1491850	11	27749725	C	2.64E-15	0.166
rs1516725	3	185824004	C	1.91E-15	0.821
rs1518170	18	40713580	C	2.37E-08	0.253
rs16851483	3	141275436	T	4.81E-08	0.161
rs16937956	11	8404501	G	9.65E-09	0.213
rs17024393	1	110154688	C	3.53E-10	0.688
rs17381664	1	78048331	C	5.31E-09	0.300
rs1846974	5	87978252	A	1.81E-08	0.652
rs1928295	9	120378483	C	3.42E-11	0.772
rs2060604	8	76650334	C	4.06E-09	0.406
rs2112347	5	75015242	G	3.15E-13	0.811
rs2237897	11	2858546	T	1.95E-11	0.652
rs2303108	19	47589895	C	8.53E-11	0.304
rs2304179	19	46122748	A	7.38E-10	0.539
rs2390669	2	169091942	C	6.01E-10	0.841
rs261966	5	95849587	C	1.99E-09	0.356
rs3127553	1	49438005	A	4.58E-10	0.474
rs35261542	6	20703952	A	9.92E-29	0.104
rs35991856	5	87978252	A	1.36E-13	0.652
rs3798519	6	50836279	C	5.22E-10	0.511
rs3817334	11	47650993	T	2.47E-11	0.567
rs3932549	9	97207669	C	2.10E-08	0.724
rs4357030	5	124316031	T	1.64E-08	0.697
rs4366055	8	95530969	C	2.23E-08	0.403
rs4409766	10	104616663	C	2.16E-10	0.703
rs4430979	2	47208	G	5.40E-10	0.586
rs4686392	3	185530290	G	1.87E-18	0.929

rs4712523	6	20657564	G	3.02E-13	0.330
rs4767475	12	112701726	G	2.61E-08	0.363
rs4790981	17	65921834	G	7.69E-09	0.408
rs4929923	11	8639200	C	9.05E-09	0.866
rs4981693	14	29680331	A	4.90E-10	0.779
rs5015933	9	128137418	C	3.66E-10	0.086
rs532504	1	177889480	A	1.06E-21	0.115
rs543874	1	177889480	G	9.61E-34	0.115
rs55934576	17	45764692	C	2.22E-08	0.180
rs6091540	20	51087862	T	2.15E-11	0.374
rs62034325	16	28559573	G	1.70E-10	0.350
rs633715	1	177852580	C	2.72E-29	0.103
rs6465468	7	95169514	T	4.98E-08	0.341
rs6548237	2	621461	C	3.55E-40	0.284
rs6567160	18	57829135	C	1.52E-32	0.243
rs663129	18	57838401	A	3.49E-34	0.235
rs66500717	8	76886813	G	1.47E-08	0.169
rs6881648	5	74997756	C	8.33E-12	0.799
rs7020996	9	22129579	T	1.50E-19	0.222
rs713586	2	25158008	C	9.02E-09	0.239
rs7138803	12	50247468	A	1.80E-17	0.273
rs7141420	14	79899454	T	1.45E-11	0.481
rs7239883	18	40147671	A	1.51E-08	0.561
rs729050	14	94109502	T	1.91E-08	0.903
rs745213	15	68060389	G	1.31E-10	0.624
rs7531118	1	72837239	C	3.90E-17	0.449
rs7903146	10	114758349	T	3.85E-11	0.970
rs8097783	18	58051294	A	2.19E-11	0.894
rs8098510	18	40796047	C	1.30E-08	0.367

rs860295	1	155850558	G	9.22E-09	0.823
rs939584	2	621558	T	6.90E-10	0.661
rs9397585	6	153396875	C	3.39E-09	0.295
rs9462027	6	34797241	A	4.60E-10	0.197
rs9568867	13	54107352	A	1.52E-13	0.990

BMI – body mass index, MR – mendelian randomization, SNP – single nucleotide polymorphism.

Table S8. Complete MR results for BMI and Gout with druggable gene targets (Inverse variance weighted *P*-value < 0.05).

Exposure	Outcome	Gene	nSNP	OR (95% CI)	*P*-value
BMI	TUBB6	TUBB6	5	0.988 (0.980–0.995)	0.0018
BMI	DPYD	DPYD	4	1.014 (1.005–1.024)	0.0037
BMI	COL6A2	COL6A2	3	0.979 (0.964–0.994)	0.0071
BMI	HLA-B	HLA-B	2	0.963 (0.936–0.991)	0.0111
BMI	GATM	GATM	3	0.973 (0.952–0.994)	0.0124
BMI	PIK3CA	PIK3CA	2	0.964 (0.937–0.993)	0.0137
BMI	CCR1	CCR1	2	0.966 (0.939–0.993)	0.0150
BMI	CDK11B	CDK11B	2	1.021 (1.004–1.038)	0.0164
BMI	CRIP2	CRIP2	4	0.986 (0.975–0.998)	0.0172
BMI	STAT6	STAT6	3	0.990 (0.981–0.998)	0.0179
BMI	CRYZ	CRYZ	2	0.987 (0.976–0.998)	0.0185
BMI	CRISPLD2	CRISPLD2	4	0.992 (0.985–0.999)	0.0209
BMI	PIK3R1	PIK3R1	2	0.975 (0.954–0.996)	0.0212
BMI	ICOS	ICOS	2	0.966 (0.937–0.995)	0.0214
BMI	COL23A1	COL23A1	3	1.036 (1.005–1.069)	0.0229
BMI	USP14	USP14	2	1.018 (1.002–1.033)	0.0232
BMI	MED12L	MED12L	2	1.041 (1.006–1.078)	0.0232
BMI	SCAP	SCAP	2	1.072 (1.010–1.139)	0.0233
BMI	TSHZ2	TSHZ2	3	1.019 (1.002–1.036)	0.0246
BMI	PLTP	PLTP	4	0.990 (0.981–0.999)	0.0246
BMI	INPP5D	INPP5D	2	1.032 (1.004–1.061)	0.0250
BMI	PPWD1	PPWD1	3	0.988 (0.978–0.998)	0.0250

BMI	NR1H3	NR1H3	5	1.027 (1.003–1.051)	0.0251
BMI	ADAMTS5	ADAMTS5	3	0.973 (0.949–0.997)	0.0274
BMI	DAPK1	DAPK1	4	1.011 (1.001–1.021)	0.0281
BMI	NIPAL2	NIPAL2	2	0.984 (0.970–0.998)	0.0285
BMI	SIGLEC9	SIGLEC9	2	1.036 (1.003–1.070)	0.0300
BMI	GPR160	GPR160	3	1.014 (1.001–1.026)	0.0315
BMI	ENGASE	ENGASE	6	1.011 (1.001–1.021)	0.0318
BMI	GARS1	GARS1	2	0.963 (0.930–0.997)	0.0326
BMI	MARK3	MARK3	2	1.029 (1.002–1.056)	0.0335
BMI	PSORS1C3	PSORS1C3	2	0.969 (0.941–0.998)	0.0353
BMI	ZNF613	ZNF613	2	1.013 (1.001–1.024)	0.0354
BMI	FOLR2	FOLR2	2	1.038 (1.003–1.076)	0.0357
BMI	ANGPT2	ANGPT2	2	1.056 (1.004–1.111)	0.0357
BMI	GPR153	GPR153	2	0.967 (0.938–0.998)	0.0359
BMI	KIR2DS4	KIR2DS4	4	1.010 (1.001–1.019)	0.0369
BMI	PRPF31	PRPF31	3	0.990 (0.982–0.999)	0.0375
BMI	ELK3	ELK3	2	0.971 (0.945–0.998)	0.0375
BMI	GFPT1	GFPT1	5	0.988 (0.977–0.999)	0.0382
BMI	TLR4	TLR4	2	0.979 (0.959–0.999)	0.0383
BMI	CHUK	CHUK	2	1.028 (1.001–1.054)	0.0385
BMI	MT2A	MT2A	2	0.986 (0.972–0.999)	0.0391
BMI	IKZF3	IKZF3	4	1.010 (1.000–1.020)	0.0404
BMI	NFKBIA	NFKBIA	2	0.984 (0.969–0.999)	0.0408
BMI	CACNG6	CACNG6	2	1.027 (1.001–1.053)	0.0417
BMI	SENP7	SENP7	7	0.994 (0.988–1.000)	0.0418
BMI	HEXB	HEXB	2	0.985 (0.970–0.999)	0.0425
BMI	ATP10A	ATP10A	4	0.989 (0.979–1.000)	0.0426
BMI	CLEC12A	CLEC12A	5	1.010 (1.000–1.021)	0.0442
BMI	MTRR	MTRR	2	0.991 (0.983–1.000)	0.0445

BMI	OXTR	OXTR	3	1.031 (1.001–1.061)	0.0454
BMI	CXCL6	CXCL6	2	1.030 (1.001–1.061)	0.0459
BMI	AARS1	AARS1	2	1.023 (1.000–1.046)	0.0461
BMI	FCRL5	FCRL5	9	0.992 (0.984–1.000)	0.0463
BMI	PPP5C	PPP5C	2	0.992 (0.983–1.000)	0.0468
BMI	CYB5R3	CYB5R3	4	1.009 (1.000–1.018)	0.0471
BMI	UGDH	UGDH	4	1.007 (1.000–1.014)	0.0474
BMI	IDH2	IDH2	2	0.975 (0.951–1.000)	0.0482
BMI	FHL2	FHL2	2	1.011 (1.000–1.023)	0.0485
BMI	PTP4A3	PTP4A3	4	0.983 (0.967–1.000)	0.0486
BMI	CXCL1	CXCL1	3	1.012 (1.000–1.024)	0.0493
HLA-DRB6	GOUT	HLA-DRB6	9	0.946 (0.907–0.987)	0.0097
UBR1	GOUT	UBR1	2	1.233 (1.045–1.454)	0.0129
JUN	GOUT	JUN	4	0.923 (0.866–0.985)	0.0157
HMGCR	GOUT	HMGCR	2	1.192 (1.033–1.375)	0.0164
PCSK5	GOUT	PCSK5	5	1.085 (1.015–1.161)	0.0174
PTGER4	GOUT	PTGER4	2	0.820 (0.695–0.969)	0.0199
FES	GOUT	FES	3	0.912 (0.844–0.986)	0.0211
FGD4	GOUT	FGD4	2	0.780 (0.631–0.964)	0.0215
BMI	PTP4A3	PTP4A3	4	0.983 (0.967–1.000)	0.0486
ARVCF	GOUT	ARVCF	2	1.242 (1.032–1.496)	0.0222
PON2	GOUT	PON2	4	1.115 (1.015–1.225)	0.0229
NUDT15	GOUT	NUDT15	2	1.401 (1.045–1.880)	0.0244
PTGFR	GOUT	PTGFR	3	0.846 (0.730–0.980)	0.0262
PPID	GOUT	PPID	2	1.433 (1.043–1.968)	0.0262
ENPP1	GOUT	ENPP1	2	1.573 (1.050–2.356)	0.0280
SEMA6A	GOUT	SEMA6A	2	1.217 (1.019–1.454)	0.0302
HSD17B11	GOUT	HSD17B11	4	0.892 (0.804–0.989)	0.0302
SLC22A5	GOUT	SLC22A5	5	0.938 (0.886–0.994)	0.0304

CYP4V2	GOUT	CYP4V2	3	1.062 (1.005–1.122)	0.0315
ALDH8A1	GOUT	ALDH8A1	3	1.243 (1.019–1.517)	0.0319
DHFR	GOUT	DHFR	3	1.101 (1.008–1.202)	0.0328
OASL	GOUT	OASL	2	0.911 (0.835–0.993)	0.0337
INVS	GOUT	INVS	3	1.110 (1.007–1.222)	0.0348
LILRA4	GOUT	LILRA4	2	0.765 (0.596–0.981)	0.0351
MTHFR	GOUT	MTHFR	5	0.930 (0.868–0.995)	0.0354
SIRPA	GOUT	SIRPA	5	0.892 (0.801–0.993)	0.0369
ULK3	GOUT	ULK3	3	1.124 (1.028–1.229)	0.0106
SPECC1L	GOUT	SPECC1L	3	1.130 (1.019–1.253)	0.0204
CYP26B1	GOUT	CYP26B1	5	0.958 (0.924–0.993)	0.0205
TUBB	GOUT	TUBB	2	1.219 (1.021–1.457)	0.0289
HLA-H	GOUT	HLA-H	3	0.889 (0.799–0.988)	0.0293
FGL2	GOUT	FGL2	3	0.848 (0.730–0.984)	0.0298
GLE1	GOUT	GLE1	2	1.126 (1.011–1.252)	0.0302
ABAT	GOUT	ABAT	5	0.926 (0.864–0.993)	0.0320
TNFRSF4	GOUT	TNFRSF4	3	0.890 (0.801–0.990)	0.0321
RXRA	GOUT	RXRA	2	0.716 (0.524–0.977)	0.0354
GUCY1A1	GOUT	GUCY1A1	2	0.795 (0.640–0.988)	0.0381
OCLN	GOUT	OCLN	2	0.818 (0.675–0.992)	0.0415
AKR1C1	GOUT	AKR1C1	3	0.845 (0.718–0.994)	0.0420
SPINT1	GOUT	SPINT1	2	0.831 (0.695–0.994)	0.0425
SPOCK2	GOUT	SPOCK2	2	1.419 (1.012–1.990)	0.0427
PGGT1B	GOUT	PGGT1B	2	0.898 (0.808–0.997)	0.0445
SPARC	GOUT	SPARC	2	0.865 (0.750–0.997)	0.0452
METTL21A	GOUT	METTL21A	4	1.070 (1.001–1.144)	0.0461
HLA-G	GOUT	HLA-G	8	0.920 (0.847–0.999)	0.0462
CYB5A	GOUT	CYB5A	2	0.895 (0.802–0.999)	0.0478
IKZF1	GOUT	IKZF1	4	1.063 (1.000–1.130)	0.0484

TRPM6	GOUT	TRPM6	3	1.075 (1.001–1.155)	0.0484
EIF4EBP2	GOUT	EIF4EBP2	2	0.895 (0.804–0.996)	0.0416
TEK	GOUT	TEK	4	0.949 (0.901–0.998)	0.0428
ARCN1	GOUT	ARCN1	2	0.864 (0.749–0.995)	0.0430
NR2C2	GOUT	NR2C2	2	1.214 (1.005–1.466)	0.0443
SOAT1	GOUT	SOAT1	3	0.923 (0.854–0.998)	0.0450
POLI	GOUT	POLI	3	0.920 (0.847–0.999)	0.0470
GLO1	GOUT	GLO1	2	0.788 (0.621–0.999)	0.0487
KCNA3	GOUT	KCNA3	2	0.826 (0.682–0.999)	0.0493
PLAGL1	GOUT	PLAGL1	4	1.070 (1.010–1.133)	0.0217
GCLC	GOUT	GCLC	3	1.128 (1.015–1.254)	0.0253
NPTX2	GOUT	NPTX2	2	0.745 (0.575–0.967)	0.0268
PRG4	GOUT	PRG4	2	1.114 (1.012–1.227)	0.0273
SEMA3A	GOUT	SEMA3A	3	0.870 (0.767–0.986)	0.0292
RPS3A	GOUT	RPS3A	2	0.812 (0.672–0.980)	0.0296
PTK2B	GOUT	PTK2B	2	0.937 (0.883–0.994)	0.0309
VIM	GOUT	VIM	3	1.127 (1.011–1.257)	0.0317
CD59	GOUT	CD59	2	0.925 (0.860–0.994)	0.0334
S1PR3	GOUT	S1PR3	2	1.091 (1.006–1.183)	0.0345
TPK1	GOUT	TPK1	3	1.059 (1.004–1.118)	0.0358
TPCN2	GOUT	TPCN2	4	1.053 (1.002–1.106)	0.0395
DERL1	GOUT	DERL1	2	0.826 (0.689–0.991)	0.0397
PPT1	GOUT	PPT1	5	1.042 (1.002–1.085)	0.0404
TXNDC12	GOUT	TXNDC12	2	0.924 (0.856–0.998)	0.0435
MTRF1L	GOUT	MTRF1L	2	0.935 (0.876–0.998)	0.0437
TUFT1	GOUT	TUFT1	2	1.222 (1.005–1.485)	0.0441
CCRL2	GOUT	CCRL2	2	1.131 (1.003–1.275)	0.0454
S100B	GOUT	S100B	4	1.046 (1.001–1.094)	0.0456
HLA-DOB	GOUT	HLA-DOB	3	0.940 (0.884–0.999)	0.0461

TRPC6	GOUT	TRPC6	3	0.918 (0.843–0.999)	0.0467
MAPK3	GOUT	MAPK3	4	0.954 (0.911–1.000)	0.0473
HSPA1B	GOUT	HSPA1B	3	1.088 (1.001–1.183)	0.0481
METAP1	GOUT	METAP1	2	0.789 (0.623–0.998)	0.0482
KRT10	GOUT	KRT10	2	0.915 (0.837–1.000)	0.0482

BMI – body mass index, MR – mendelian randomization, OR – odd ratio, CI – confidence interval, SNP – single nucleotide polymorphism.

Table S9. MR analysis of genetically predicted druggable gene targets on other types of gout risk (Inverse variance weighted *P*-value < 0.05).

Exposure	Outcome	Gene	nSNP	OR (95% CI)	*P*-value
BMI	Other Gout	POMT2	3	3.102 (1.273–7.557)	0.0127
BMI	Other Gout	IFIT2	2	0.092 (0.013–0.659)	0.0176
BMI	Other Gout	NCR3	2	8.455 (1.390–51.436)	0.0205
BMI	Other Gout	PDGFD	3	0.460 (0.235–0.899)	0.0231
BMI	Other Gout	RPS23	5	1.599 (1.066–2.399)	0.0233
BMI	Other Gout	ALOX5AP	2	1.976 (1.096–3.560)	0.0235
BMI	Other Gout	SLC6A16	2	2.669 (1.135–6.275)	0.0244
BMI	Other Gout	KCNMB1	4	1.783 (1.069–2.973)	0.0267
BMI	Other Gout	ITGA7	2	4.589 (1.146–18.373)	0.0314
BMI	Other Gout	RAB40B	2	3.502 (1.119–10.963)	0.0314
BMI	Other Gout	ACP3	3	2.078 (1.067–4.049)	0.0316
BMI	Other Gout	GZMK	2	2.646 (1.088–6.434)	0.0319
BMI	Other Gout	RAB5A	3	2.308 (1.067–4.993)	0.0335
BMI	Other Gout	FZD6	5	1.723 (1.034–2.869)	0.0366
BMI	Other Gout	IGLV7-46	3	2.971 (1.065–8.289)	0.0375
BMI	Other Gout	MAN2C1	6	1.437 (1.018–2.027)	0.0391
BMI	Other Gout	IFI27	2	0.497 (0.254–0.971)	0.0406
BMI	Other Gout	SMOX	3	1.744 (1.024–2.970)	0.0408
BMI	Other Gout	SEMA3A	3	2.902 (1.042–8.081)	0.0414
BMI	Other Gout	CD33	2	0.521 (0.278–0.976)	0.0416
BMI	Other Gout	HMBS	3	1.815 (1.021–3.227)	0.0421
BMI	Other Gout	TMEM123	2	2.676 (1.034–6.928)	0.0425

BMI	Other Gout	PDE9A	3	0.579 (0.341–0.983)	0.0430
BMI	Other Gout	PADI4	2	0.519 (0.275–0.980)	0.0430
BMI	Other Gout	AKR1B1	4	1.872 (1.019–3.438)	0.0433
BMI	Other Gout	NLRP1	4	1.817 (1.012–3.264)	0.0456
BMI	Other Gout	CCN2	2	0.453 (0.208–0.987)	0.0461
BMI	Other Gout	CCR6	2	0.480 (0.233–0.989)	0.0467
BMI	Other Gout	MMP17	2	4.145 (1.013–16.958)	0.0479
BMI	Other Gout	HOXB3	2	1.779 (1.004–3.153)	0.0486
BMI	Other Gout	HOXB4	2	1.662 (1.003–2.756)	0.0487
BMI	Other Gout	GCNT1	3	0.450 (0.204–0.997)	0.0490
BMI	Other Gout	PEPD	2	0.763 (0.619–0.939)	0.0107
BMI	Other Gout	UGGT2	4	1.153 (1.026–1.295)	0.0163
BMI	Other Gout	TNNC2	5	1.146 (1.021–1.286)	0.0204
BMI	Other Gout	NDUFS5	5	0.907 (0.832–0.989)	0.0263
BMI	Other Gout	MSLN	3	1.326 (1.031–1.704)	0.0277
BMI	Other Gout	SLC22A5	4	0.910 (0.837–0.990)	0.0279
BMI	Other Gout	PRR4	3	1.135 (1.013–1.273)	0.0296
BMI	Other Gout	SLC4A8	2	0.778 (0.620–0.977)	0.0305
BMI	Other Gout	GPR42	2	0.721 (0.536–0.970)	0.0305
BMI	Other Gout	CD59	2	0.862 (0.753–0.987)	0.0315
BMI	Other Gout	MBNL1	4	1.122 (1.010–1.246)	0.0322
BMI	Other Gout	IPO11	2	0.737 (0.557–0.977)	0.0335
BMI	Other Gout	CD9	3	0.909 (0.832–0.993)	0.0336
BMI	Other Gout	CEP43	2	0.875 (0.771–0.994)	0.0408
BMI	Other Gout	ACAT2	2	0.852 (0.730–0.994)	0.0422
BMI	Other Gout	HLA-DQB1	2	0.895 (0.804–0.997)	0.0430
BMI	Other Gout	TNK2	3	1.347 (1.009–1.798)	0.0431
BMI	Other Gout	AIFM2	2	1.186 (1.005–1.400)	0.0438
BMI	Other Gout	LGALS8	3	0.854 (0.731–0.997)	0.0450

BMI	Other Gout	C4A	4	0.880 (0.776–0.998)	0.0460
BMI	Other Gout	CSE1L	2	1.168 (1.003–1.360)	0.0461
BMI	Other Gout	PLAGL1	4	1.116 (1.001–1.244)	0.0470
BMI	Other Gout	IGFBP4	2	1.193 (1.002–1.421)	0.0475
BMI	Other Gout	TMEM43	2	1.223 (1.002–1.493)	0.0480
BMI	Other Gout	ZNF165	2	1.544 (1.002–2.379)	0.0488
BMI	Other Gout	ABO	3	0.659 (0.481–0.903)	0.0096
BMI	Other Gout	ADCK1	3	0.640 (0.451–0.910)	0.0128
BMI	Other Gout	BBS2	7	1.442 (1.080–1.925)	0.0131
BMI	Other Gout	FPGS	2	0.240 (0.077–0.746)	0.0136
BMI	Other Gout	LGALS9	4	0.681 (0.499–0.930)	0.0157
BMI	Other Gout	ADARB1	2	1.592 (1.086–2.332)	0.0171
BMI	Other Gout	SLC24A4	4	1.443 (1.067–1.953)	0.0173
BMI	Other Gout	ABAT	4	0.672 (0.477–0.947)	0.0230
BMI	Other Gout	STK24	2	2.188 (1.111–4.307)	0.0235
BMI	Other Gout	ITM2B	2	1.693 (1.067–2.688)	0.0255
BMI	Other Gout	DHFR	3	1.540 (1.053–2.253)	0.0260
BMI	Other Gout	CDC7	4	0.709 (0.523–0.963)	0.0275
BMI	Other Gout	RAC2	2	1.605 (1.051–2.452)	0.0287
BMI	Other Gout	RPA1	4	1.413 (1.025–1.947)	0.0349
BMI	Other Gout	UBA6	2	2.991 (1.070–8.359)	0.0367
BMI	Other Gout	JAK2	2	1.666 (1.032–2.689)	0.0367
BMI	Other Gout	CDKAL1	3	0.624 (0.400–0.973)	0.0372
BMI	Other Gout	NUDT2	8	0.803 (0.651–0.990)	0.0395
BMI	Other Gout	SPTLC3	2	0.324 (0.111–0.950)	0.0401
BMI	Other Gout	MAP3K20	3	0.693 (0.486–0.986)	0.0418
BMI	Other Gout	IRF6	2	0.716 (0.519–0.989)	0.0425
BMI	Other Gout	BPHL	2	0.442 (0.200–0.977)	0.0436
BMI	Other Gout	PPIL1	2	2.045 (1.020–4.101)	0.0438

BMI	Other Gout	RNASE6	4	0.783 (0.616–0.994)	0.0444
BMI	Other Gout	SULT2B1	2	1.925 (1.014–3.657)	0.0453
BMI	Other Gout	DHRS4	2	2.694 (1.020–7.116)	0.0455
BMI	Other Gout	NCOA1	2	1.921 (1.011–3.651)	0.0463
BMI	Other Gout	HSPA1L	2	3.354 (1.017–11.058)	0.0468
BMI	Other Gout	S1PR3	2	1.598 (1.006–2.540)	0.0473
BMI	Other Gout	FCGR3B	2	0.281 (0.080–0.986)	0.0475
BMI	Other Gout	NENF	3	0.300 (0.113–0.802)	0.0163
BMI	Other Gout	PPAT	3	0.143 (0.029–0.703)	0.0166
BMI	Other Gout	DBH	2	0.156 (0.033–0.726)	0.0179
BMI	Other Gout	DOCK2	3	0.423 (0.201–0.887)	0.0228
BMI	Other Gout	HLA-F	9	0.681 (0.489–0.949)	0.0234
BMI	Other Gout	FKBP1A	4	0.504 (0.277–0.916)	0.0245
BMI	Other Gout	ACACB	2	0.590 (0.371–0.939)	0.0260
BMI	Other Gout	SCNN1A	2	9.163 (1.280–65.595)	0.0274
BMI	Other Gout	FCGRT	2	2.850 (1.095–7.421)	0.0320
BMI	Other Gout	PSMD6	2	16.390 (1.213–221.464)	0.0353
BMI	Other Gout	CAST	3	0.526 (0.289–0.957)	0.0354
BMI	Other Gout	MPST	2	0.102 (0.012–0.857)	0.0355
BMI	Other Gout	IRF2	2	0.314 (0.106–0.928)	0.0362
BMI	Other Gout	MAP3K5	2	0.433 (0.197–0.951)	0.0370
BMI	Other Gout	PPIL2	2	0.315 (0.106–0.935)	0.0374
BMI	Other Gout	NUCB1	4	1.844 (1.024–3.320)	0.0414
BMI	Other Gout	PEAR1	2	0.308 (0.098–0.963)	0.0428
BMI	Other Gout	GNS	2	0.341 (0.119–0.976)	0.0450
BMI	Other Gout	EEF2	2	0.116 (0.014–0.964)	0.0462
BMI	Other Gout	NR1D2	2	0.279 (0.079–0.989)	0.0480
BMI	Other Gout	NUDT15	2	0.105 (0.011–0.992)	0.0492

BMI – body mass index, MR – mendelian randomization, OR – odd ratio, CI – confidence

interval, SNP – single nucleotide polymorphism.

Table S10. Colocalization analysis of genetically predicted BMI with Gout and other types of Gout for druggable gene targets (PP.H4 > 0.75).

Analysis	Gene	PP.H0	PP.H1	PP.H2	PP.H3	PP.H4
BMI & Gout	HMGCR	1.77E-54	3.08E-11	1.85E-46	2.22E-03	0.998
BMI & Gout	MAPK3	0	2.69E-16	0	1.07E-02	0.989
BMI & other Gout	HOXB4	0	1.55E-02	0	3.15E-02	0.953
BMI & Gout	GATM	0	1.33E-01	0	3.14E-02	0.835
BMI & other Gout	GATM	0	1.33E-01	0	3.14E-02	0.835
BMI & Gout	SEMA6A	6.89E-45	1.14E-01	3.15E-45	5.12E-02	0.835
BMI & Gout	INVS	2.51E-201	1.35E-01	1.90E-201	1.01E-01	0.764
BMI & other Gout	CCR6	1.29E-141	2.20E-01	1.40E-142	2.31E-02	0.757

BMI – body mass index.

Figure S1. The workflow diagram of this study. NHANES – national health and nutrition examination survey, BMI – body mass index, MR – mendelian randomization, IVW – inverse-variance weighted, OR – odd ratio, LD – linkage disequilibrium.

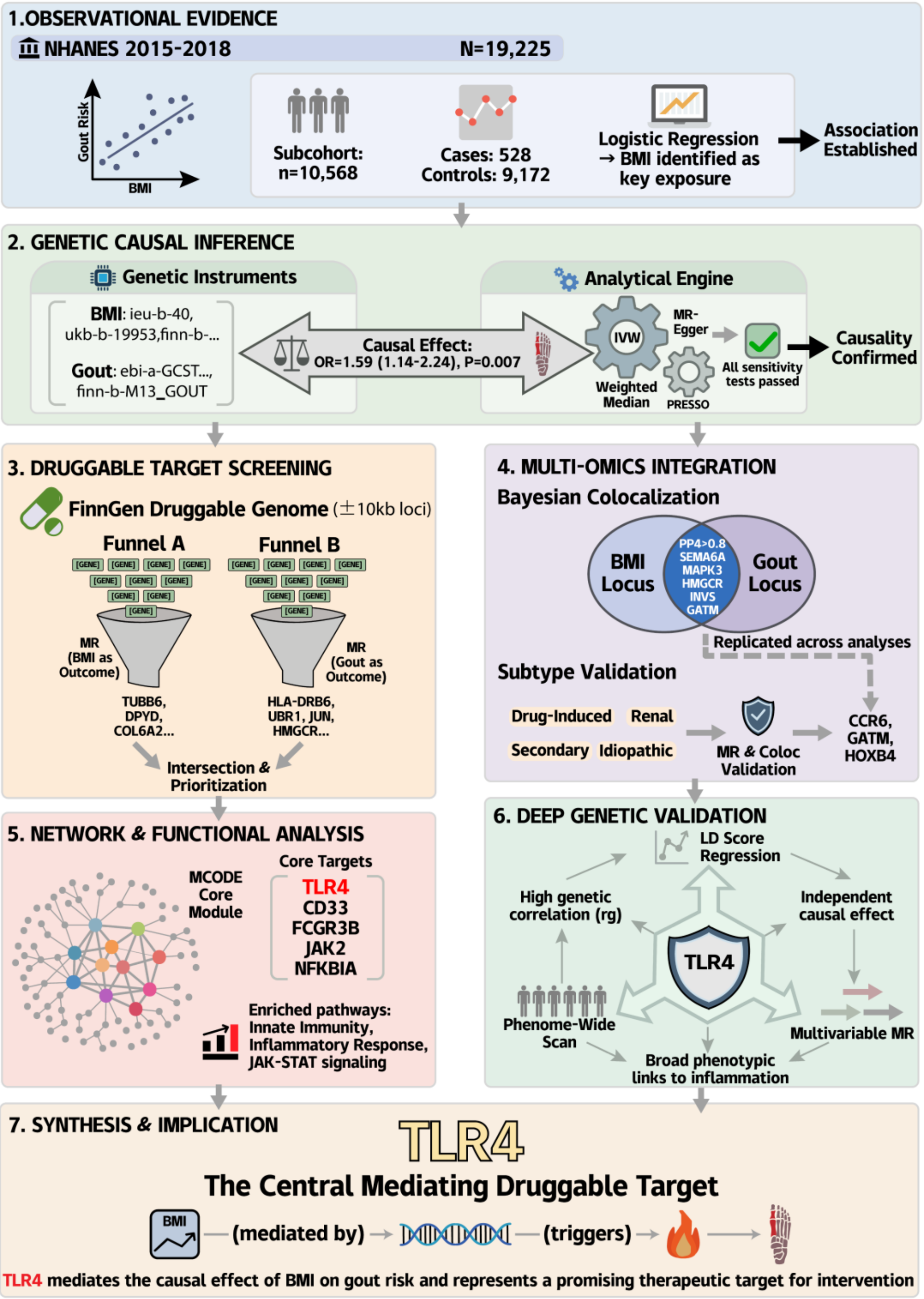

Figure S2. DAG depicting the hypothesized causal framework and mediation pathways. Path a (BMI to gout): direct causal effect of adiposity on gout risk, estimated by two-sample Mendelian randomization (MR). Path b (gene to BMI): effect of genetically proxied perturbation of a

druggable gene on BMI. Path c (gene to gout): effect of the same gene on gout risk. Evidence for both paths b and c is consistent with a candidate mediating role of the gene product. Path c' (gene to gout, adjusted for BMI): BMI-independent effect of the gene on gout, estimated by multivariable MR. Dashed arrows denote potential confounding pathways (age, gender, race, etc.) that may influence both BMI and gout. BMI – body mass index, MR – mendelian randomization.

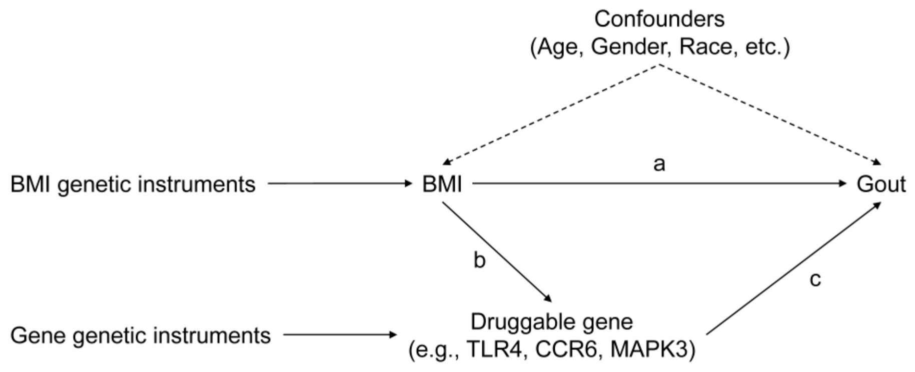

Path c′ (not shown) represents the BMI-independent effect of the gene on gout tested in multivariable MR.

Figure S3. Dose-response relationships between BMI and the risk of gout across study cycles and population subgroups in the NHANES cohort. NHANES – national health and nutrition examination survey, RCS – restricted cubic spline, OR – odd ratio.

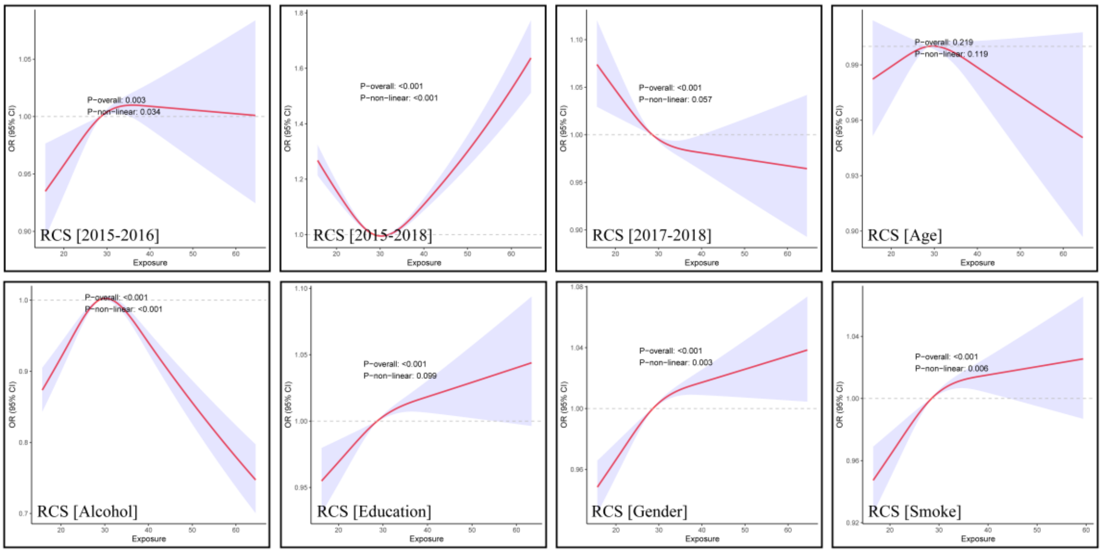

Figure S4. Subgroup analysis of the association between continuous BMI and gout risk in the NHANES cohort. CI – confidence interval.

Variable	Group	Beta (95% CI)	pvalue	Interaction_pvalue
Gender	Female	0.045 (0.027 to 0.063)	<0.001	0.595
	Male	0.050 (0.035 to 0.065)	<0.001	
Age	<=65	0.042 (0.023 to 0.060)	<0.001	0.220
	>65	0.065 (0.038 to 0.092)	<0.001	
Race	Mexican American	0.055 (0.012 to 0.097)	0.018	0.114
	Non−Hispanic Black	0.012 (−0.006 to 0.031)	0.194	
	Non−Hispanic White	0.050 (0.033 to 0.066)	<0.001	
	Other Hispanic	0.045 (−0.006 to 0.097)	0.097	
	Other Race − Including Multi−Racial	0.047 (0.014 to 0.079)	0.008	
Education	Less than 9th grade	0.055 (0.012 to 0.097)	0.017	0.024
	9−11th grade	0.041 (0.014 to 0.068)	0.006	
	High school graduate	0.009 (−0.011 to 0.030)	0.385	
	Some college or AA degree	0.050 (0.032 to 0.068)	<0.001	
	College graduate or above	0.056 (0.028 to 0.084)	<0.001	
Alcohol	No	0.056 (0.031 to 0.082)	<0.001	0.401
	Yes	0.041 (0.026 to 0.056)	<0.001	
Folic	Q1	0.064 (0.040 to 0.087)	<0.001	0.119
	Q2	0.023 (−0.002 to 0.047)	0.080	
	Q3	0.027 (−0.010 to 0.064)	0.159	
	Q4	0.056 (0.033 to 0.079)	<0.001	
Smoke	No	0.044 (0.023 to 0.066)	<0.001	0.760
	Yes	0.040 (0.024 to 0.056)	<0.001	

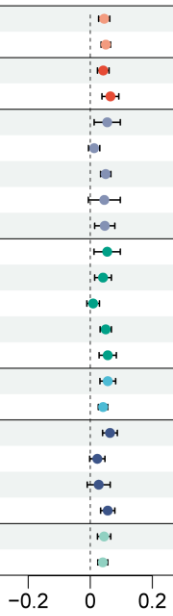

Figure S5. MR diagnostics for three key BMI-gout analyses. MR – mendelian randomization, BMI – body mass index, SNP – single nucleotide polymorphism.

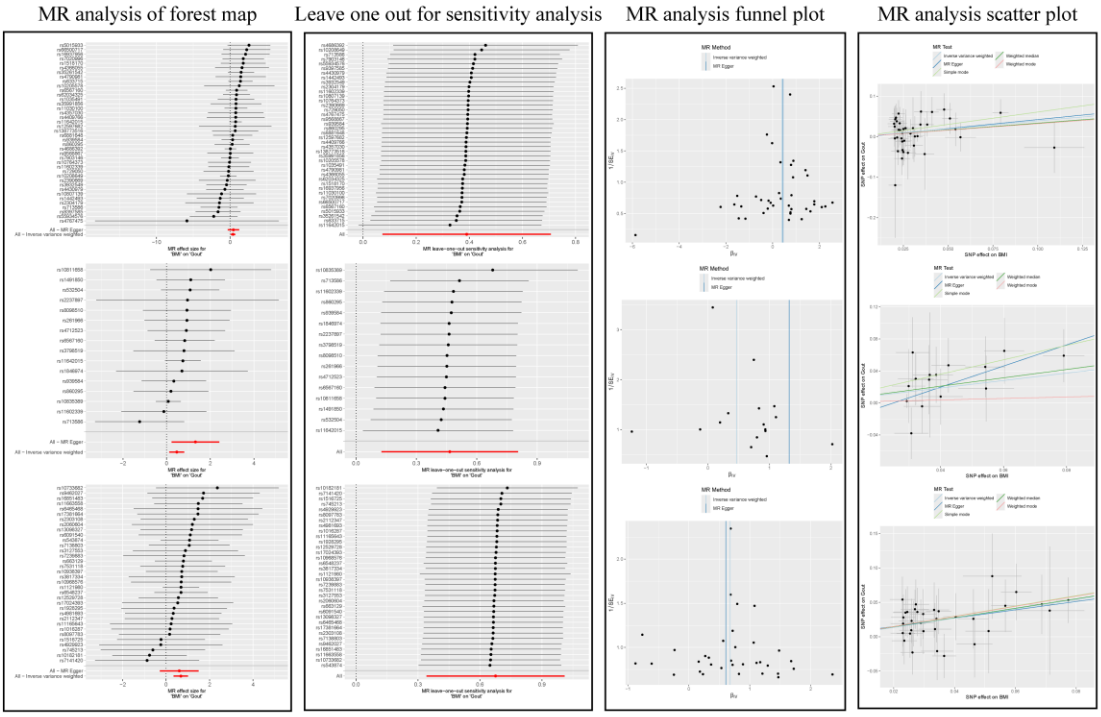

Figure S6. Sensitivity analyses and consistency of MR estimates for BMI and gout. MR – mendelian randomization, BMI – body mass index.

exposure	outcome	nsnp	method	pval	OR(95% CI)
BMI	Gout	16	MR Egger	**0.032**	3.746 (1.260 to 11.140)
		16	Weighted median	**0.032**	1.685 (1.047 to 2.714)
		16	Inverse variance weighted	**0.007**	1.593 (1.135 to 2.236)
		16	Simple mode	0.057	2.453 (1.044 to 5.764)
		16	Weighted mode	0.723	1.097 (0.664 to 1.812)
		40	MR Egger	0.272	1.557 (0.715 to 3.392)
		40	Weighted median	0.148	1.397 (0.888 to 2.198)
		40	Inverse variance weighted	**0.016**	1.477 (1.076 to 2.028)
		40	Simple mode	0.122	1.838 (0.864 to 3.909)
		40	Weighted mode	0.207	1.379 (0.844 to 2.253)
		34	MR Egger	0.193	1.831 (0.751 to 4.462)
		34	Weighted median	**0.006**	1.993 (1.221 to 3.252)
		34	Inverse variance weighted	**<0.001**	1.965 (1.407 to 2.745)
		34	Simple mode	0.091	2.106 (0.911 to 4.870)
		34	Weighted mode	**0.023**	2.086 (1.139 to 3.819)

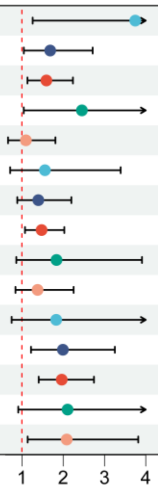

Figure S7. Bayesian colocalization analysis identifies shared genetic loci between BMI and gout. GWAS – genome-wide association study, pQTL – protein quantitative trait loci.

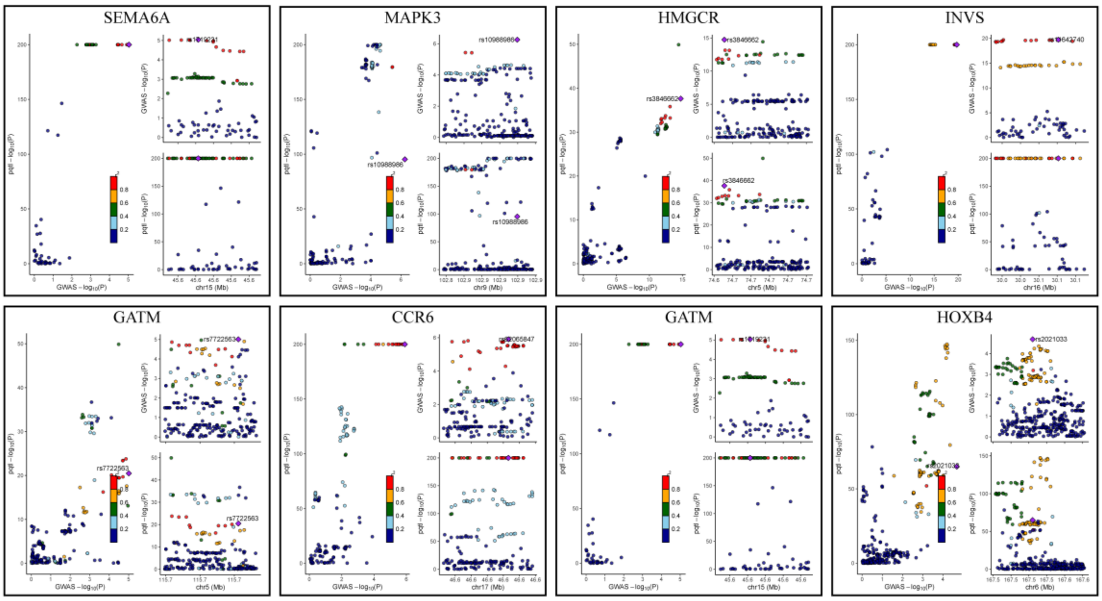

Figure S8. PPI network and functional enrichment analysis of druggable gene targets. KEGG – kyoto encyclopedia of genes and genomes.

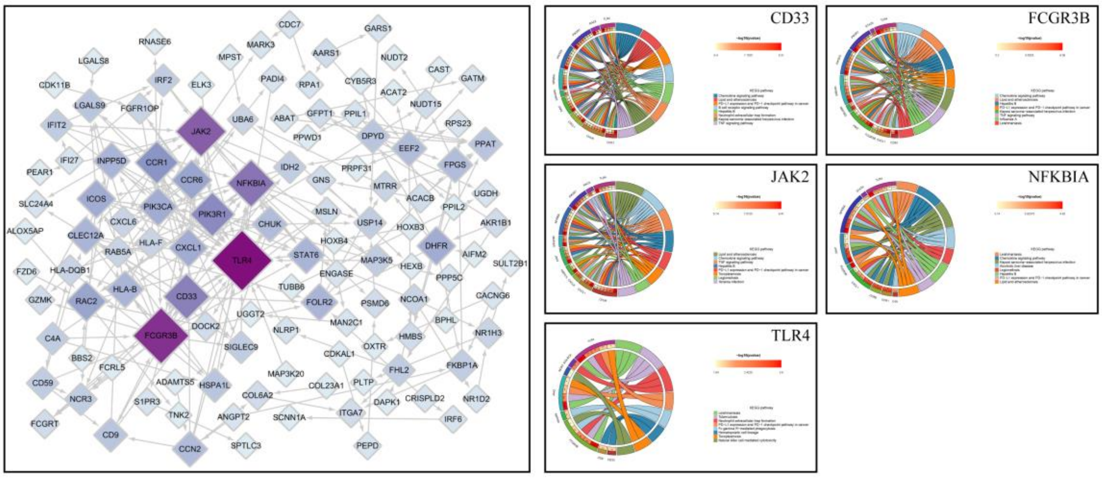

Figure S9. MVMR analysis of TLR4. GWAS – genome-wide association study.

Keratoderma (acquired): 87 cases, 403,875 controls

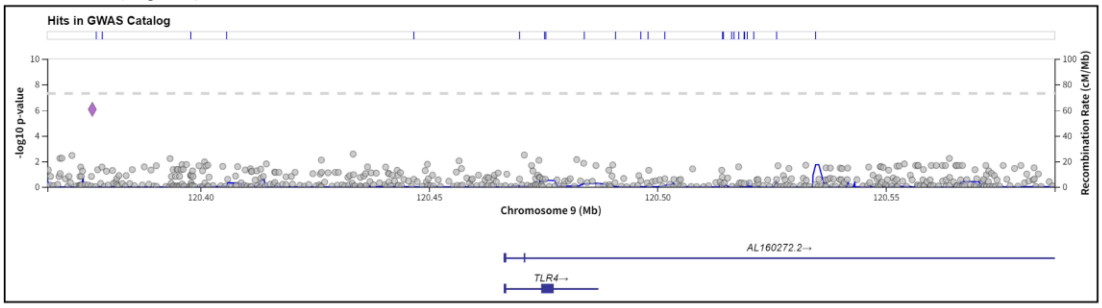

Other biliary tract disease: 3892 cases, 391,307 controls

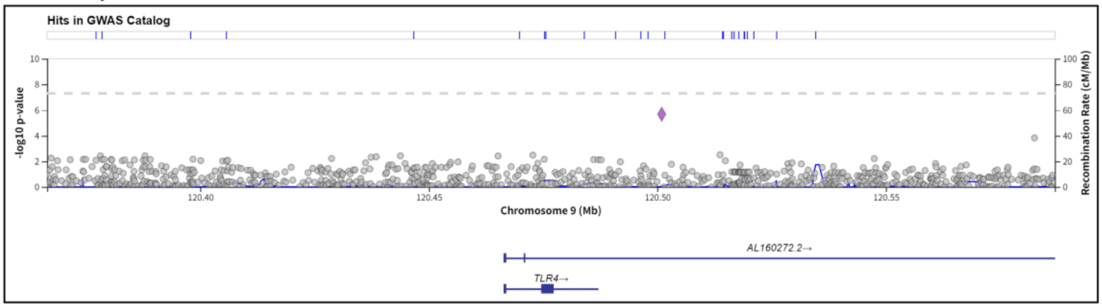

Nausea and vomiting: 11,706 cases, 397,255 controls

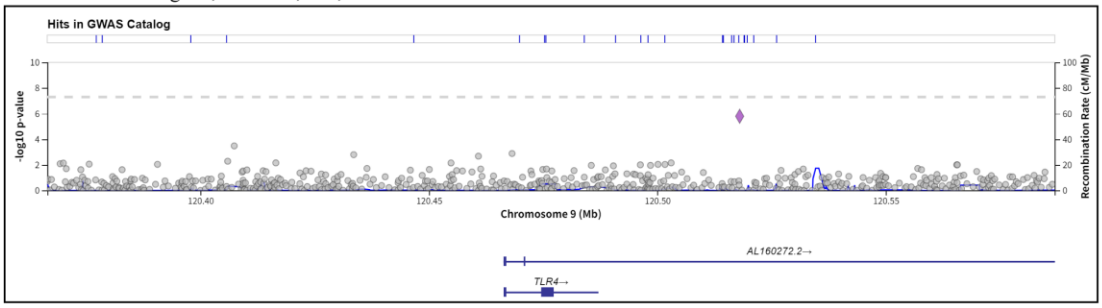

Vascular disorders of kidney/hypertrophy: 77 cases, 397,602 controls

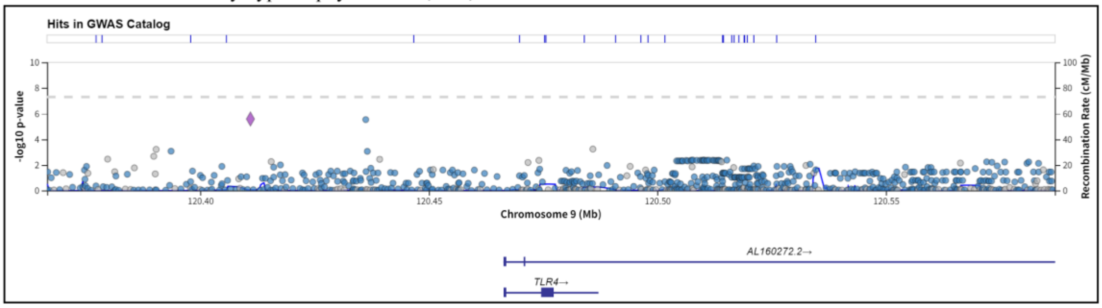

Figure S10. PheWAS analysis of manhattan plot.

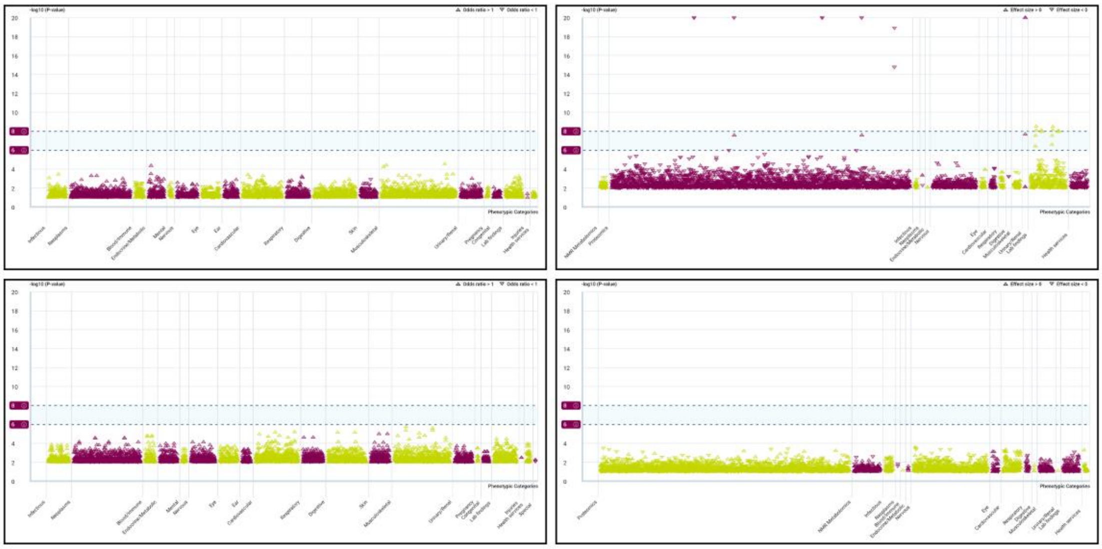

Supplement: Online Supplementary Document [file jogh-16-04268-s001.pdf]
